# The Dissection of SNAREs Reveals Key Factors for Vesicular Trafficking to the Endosome-like Compartment and Apicoplast via the Secretory System in Toxoplasma gondii

**DOI:** 10.1128/mBio.01380-21

**Published:** 2021-08-03

**Authors:** Shinuo Cao, Juan Yang, Jiawen Fu, Heming Chen, Honglin Jia

**Affiliations:** a State Key Laboratory of Veterinary Biotechnology, Harbin Veterinary Research Institute, Chinese Academy of Agricultural Sciences, Harbin, People’s Republic of China; UT Southwestern Medical Center; University of Arizona

**Keywords:** SNARE, TgVAMP4-2, TgStx19, TgGS27, TgTrs85, *Toxoplasma gondii*

## Abstract

Vesicular trafficking is a fundamental cellular process involved in material transport in eukaryotes, but the diversity of the intracellular compartments has prevented researchers from obtaining a clear understanding of the specific functions of vesicular trafficking factors, including SNAREs, tethers, and Rab GTPases, in *Apicomplexa*. In this study, we analyzed the localization of SNAREs and investigated their roles in vesicular trafficking in Toxoplasma gondii. Our results revealed the specific localizations of SNAREs in the endoplasmic reticulum (ER) (T. gondii Stx18 [TgStx18] and TgStx19), Golgi stacks (TgGS27), and endosome-like compartment (TgStx10 and TgStx12). The conditional ablation of ER- and Golgi-residing SNAREs caused severe defects in the secretory system. Most importantly, we found an R-SNARE (TgVAMP4-2) that is targeted to the apicoplast; to our knowledge, this work provides the first information showing a SNARE protein on endosymbiotic organelles and functioning in vesicular trafficking in eukaryotes. Conditional knockout of TgVAMP4-2 blocked the entrance of TgCPN60, TgACP, TgATrx2, and TgATrx1 into the apicoplast and interfered with the targeting of TgAPT1 and TgFtsH1 to the outermost membrane of the apicoplast. Together, our findings revealed the functions of SNAREs in the secretory system and the transport of nucleus-encoded proteins to an endosymbiotic organelle in a model organism of *Apicomplexa*.

## INTRODUCTION

The phylum *Apicomplexa* consists of obligate intracellular parasites, including *Plasmodium* spp. and Toxoplasma gondii, which are known to parasitize vertebrates and invertebrate hosts and thereby cause a tremendous disease burden; thus, these parasites are of great medical importance worldwide (1). T. gondii, an obligate intracellular alveolate, is a leading cause of one of the most widespread infections in humans and other warm-blooded animals and has been recognized as an important opportunistic pathogen of fetuses and immunocompromised patients (2).

*Apicomplexa* comprise single-celled eukaryotes with diverse endomembrane systems. In T. gondii, the endoplasmic reticulum (ER) is highly reduced, and the nuclear envelope provides a substantial fraction of the ER volume (3). A single Golgi complex with limited stacks is closely juxtaposed with the ER (4). At the apical end, T. gondii assembles a polarized apical complex that contains secretory organelles, including micronemes and rhoptries (5). These organelles release numerous components that mediate the invasion process and survival in host cells. Moreover, most *Apicomplexa* species, including T. gondii, contain a nonphotosynthetic plastid called the apicoplast, which houses metabolic pathways essential for parasite survival (6, 7). Certainly, coordination among groups of vesicular trafficking machinery is needed for organelle maturation and the transport of materials to specific compartments in this organism (8).

All processes through which rhoptry proteins (RONs and ROPs), microneme proteins (MICs), and nucleus-encoded apicoplast-targeted (NEAT) proteins are transported are associated with the secretory pathway in T. gondii (9). The secretory system of T. gondii is markedly polarized, because this parasite has only a singular Golgi stack (4). The current model suggests that the *trans*-Golgi network (TGN) of this parasite resembles that of plants and acts as a protein-sorting platform for secretory and endocytic cargoes (10). MICs are sorted by the sortilin, T. gondii SORTLR (TgSORTLR) at the TGN, and then delivered to the endosome-like compartment (ELC) for further processing (11). A retromer complex (Vps35-Vps26-Vps29) is needed to recycle this receptor at the ELC (12). ELC is decorated with Rab5a, Rab5c, and Rab7 and appears to have both secretion and endocytosis functions (13–15). In addition, ELC is essential for the assembly of the inner membrane complex (IMC) (14, 16, 17). Together with tethering factors (HOPS and COVET) and an SM protein (TgVps45), a soluble N-ethylmaleimide-sensitive factor (NSF) attachment protein receptor (SNARE) complex, including TgStx6, TgStx16, and TgVtila, is localized at the TGN to ELC and required for the biogenesis of secretory organelles (16, 18, 19). Little is known about the vesicular trafficking factors involved in early and intra-Golgi trafficking. More importantly, the pathway responsible for NEAT protein transport to the apicoplast remains unclear. The ER membrane is believed to fuse with the outermost membrane of the symbionts in most other chromalveolates, such as diatoms (20). In contrast, increasing evidence suggests that a vesicular trafficking pathway exists between the ER and apicoplast and mediates NEAT protein transport (21–23). However, none of the vesicular trafficking factors involved in this route have been identified.

Protein and lipid transport between organelles of the endomembrane system relies on conserved machinery of vesicular trafficking in eukaryotes (24). Cargoes are first packaged into vesicles at donor organelles and transported via the cytoskeleton to the destination. The vesicles are then recognized and fused with target membranes by the interplay of tethering factors, SM proteins, Rab GTPases, and SNARE proteins (25). Numerous SNAREs and tethering factors are involved in the ER and Golgi transport in mammalian cells (25). SNAREs play central roles in driving membrane interactions and fusion events by forming a fusion event of two opposing membranes. The crystal structure analysis of SNARE complexes led to the classification of SNAREs into R and Q groups (26) and suggested that a SNARE complex should contain three Q-SNARE motifs and one R-SNARE motif. Tethering factors are known to be involved in SNARE complex assembly and, thus, play an active role in the specificity and speed of the fusion (27–29). These tethers are classified into two groups: homodimeric long coiled-coil proteins and multisubunit tethering complexes (MTCs). MTCs including Dsl1, conserved oligomeric Golgi (COG), and Golgi-associated retrograde protein (GARP) are associated with ER and Golgi trafficking. The multisubunit transport protein particle (TRAPP) complexes (I, II, and III) are also involved in the Golgi trafficking. These complexes are distinct tethering factors because they act as guanine nucleotide exchange factors (GEFs) for the Rab GTPases (30, 31). TRAPP III has been found at different sites, including COPII vesicles, the Golgi complex, and autophagic structures (31).

In this study, we aimed to understand the roles of SNAREs in vesicular trafficking in T. gondii. Using an auxin-inducible degron-based system, we investigated the functions of SNAREs in vesicular trafficking at the ER, Golgi complex, ELC, and apicoplast. We found that a distinctive Qc-SNARE, TgStx19, coordinates with a conserved Qa-SNARE, TgStx18, and plays essential roles in the ER. The TgGS27 and TgTrs85 (a specific subunit of the TRAPP III complex) are conserved and crucial for Golgi biogenesis. We also found that disruption of TgGS27 and TgTrs85 induces an accumulation of lipids and interferes with lipid metabolism in T. gondii. Three SNAREs, TgStx10, TgStx12, and TgVAMP4-1, are localized explicitly at the ELC and mediate multiple functions of this organelle. More importantly, our results revealed that the R-SNARE TgVAMP4-2 is localized on the outermost membrane of the apicoplast and governs the vesicular transport of NEAT proteins. To the best of our knowledge, our report provides the first characterization of a SNARE on the outermost membrane of an endosymbiotic organelle in eukaryotic cells. It provides strong and solid evidence showing that the transport of NEAT proteins to endosymbiotic plastids is mediated by the vesicular trafficking pathway.

## RESULTS

### TgStx18 and TgStx19 are localized at the ER and are critical for parasite viability.

**FIG 1:**
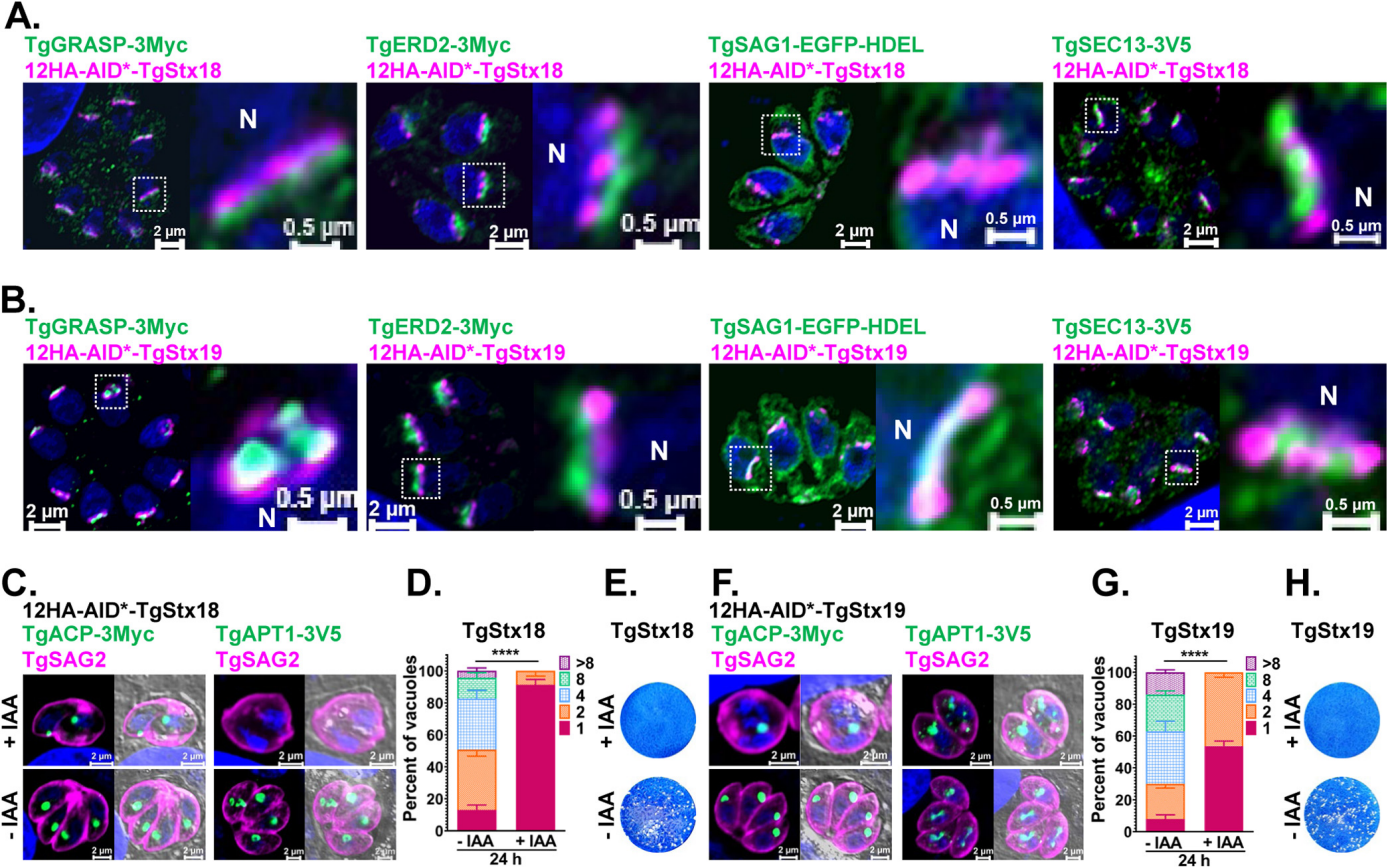
Depletion of TgStx18 and Stx19 severely affected the viability of the parasites and disturbed the trafficking of NEAT proteins to the apicoplast. (A) Immunofluorescence analysis of the 12HA-AID*-TgStx18 strain coexpressing TgGRASP-3Myc, TgERD2-3Myc, TgSAG1-EGFP-HDEL, and TgSEC13-3V5 as *cis*-Golgi and ER markers, respectively. (B) Immunofluorescence analysis as in panel A, except now with the 12HA-AID*-TgStx19 strain. (C and F) Conditional ablation of TgStx18 and TgStx19 affected the trafficking of TgACP-3Myc and TgAPT1-3V5 to the apicoplast. Parasites were added to HFF cells, allowed to invade for 3 h under normal growth conditions, and then grown in the absence or presence of IAA for 16 h. (D and G) Replication of 12HA-AID*-TgStx18 and 12HA-AID*-TgStx19 strains after growth in the absence or presence of IAA for 24 h. (E and H) Plaque assay measuring the growth of 12HA-AID*-TgStx18 and 12HA-AID*-TgStx19 parasites and parental strains for 8 days in the presence or absence of IAA.

In our previous study, 26 SNAREs were isolated from the ToxoDB database (16). In the first round of the analysis, we transiently transfected plasmids expressing tagged proteins and detected the localization by immunofluorescence assay (see Fig. S1 and Table S1 in the supplemental material). However, the accurate localization of SNAREs could not be observed in overexpressed conditions. We then constructed a tag consisting of 12HA fused to a truncated AID sequence (12HA-AID*) (32) and inserted the tag at the N terminus of the genes in the genomic loci. Most of the SNAREs allowed integration of this large tag and are responsible for auxin regulation (Fig. S2B, D, F, I, K, and M, S3B, and S4B, D, H, J, M, and O). In mammalian cells, Stx18 (Qa) is a SNAP (soluble NSF attachment protein) receptor localized in an ER-localized SNARE. Together with Sec20 (Qb), Sec22b (R), and Use1 (Qc, also called Slt1), Stx18 is involved in COPI-mediated retrograde transport from the Golgi complex to the ER (33). In T. gondii, Stx18, Sec20, and Sec22b could be recognized based on a BLAST search of human proteins but no clear orthologue was found for Use1. The localization of a putative Stx18 (TgStx18) was examined by an immunofluorescence antibody test (IFAT) and observed using an Airyscan detector. Conserved by its orthologues, TgStx18 was close to *cis*-Golgi markers (TgGRASP and TgERD2) (Fig. 1A and B). TgStx18 partially colocalized with an ER marker (TgSAG1-EGFP-HDEL) but did not merge with an ER exit site (ERES) protein (TgSec13) (Fig. 1A and B). These data are consistent with the literature that Stx18 is less associated with ERES but localized at ER arriving sites (ERAS) (34).

10.1128/mBio.01380-21.1 FIG S1 Localization of SNAREs encoded in T. gondii. Localization was detected by transfection with plasmids to express FKBP-HA-tagged SNAREs or SNAREs endogenously tagged with 12HA-AID*, as indicated in the figure. Download FIG S1, TIF file, 2.8 MB. Copyright © 2021 Cao et al. 2021 Cao et al. https://creativecommons.org/licenses/by/4.0/ This content is distributed under the terms of the Creative Commons Attribution 4.0 International license.

10.1128/mBio.01380-21.2 FIG S2 Generation of the 12HA-AID*-TgStx19, 12HA-AID*-TgStx18, and 12HA-AID*-TgGS27 strains and characterization of TgStx19, TgStx18, and TgGS27. (A, E, and J) Schematic representation of the AID*-based system used to inactivate TgStx19, TgStx18, and TgGS27 conditionally. (B, F, and K) IFA analysis of TgStx19, TgStx18, and TgGS27 (green) in 12HA-AID*-TgStx19, 12HA-AID*-TgStx18, and 12HA-AID*-TgGS27 parasites cultured in the presence of 500 nM IAA or vehicle (ethanol). (C, G, and L) Genomic PCR analysis using the indicated primers confirmed homologous recombination of the TgStx19, TgStx18, and TgGS27 genomic loci. (D, I, and M) Western blot analysis of 12HA-AID*-TgStx19, 12HA-AID*-TgStx18, and 12HA-AID*-TgGS27 parasites cultured in the presence of 500 nM IAA or vehicle (ethanol) for 0, 4, 8, and 16 h. (H) Conditional ablation of 12HA-AID*-TgStx18 induced the accumulation of lipid droplets in the parasitic cytoplasm. Parasites were added to HFF cells to invade for 3 h under normal growth conditions and then grown in the absence or presence of IAA for 16 h. Download FIG S2, TIF file, 2.6 MB. Copyright © 2021 Cao et al. 2021 Cao et al. https://creativecommons.org/licenses/by/4.0/ This content is distributed under the terms of the Creative Commons Attribution 4.0 International license.

10.1128/mBio.01380-21.3 FIG S3 Generation of the TgTrs85-mAID-3HA strain and characterization of TgTrs85. (A) Schematic representation of the mAID-based system used to deplete TgTrs85 conditionally. (B) Immunofluorescence analysis of TgTrs85-mAID-3HA (green) in TgTrs85-mAID-3HA parasites cultured in the presence of 500 nM IAA or the vehicle (EtOH) for 16 h. (C) Genomic PCR analysis using the indicated primers confirmed homologous recombination of the TgTrs85 genomic locus. The primers used are listed in Table S3. (D and E) The assembly of TgIMC and TgβTubulin in daughter parasites was detected in the TgTrs85 mutant. TgTrs85-mAID-3HA parasites were cultured in the presence or absence of IAA for 48 h. (F and G) Depletion of TgTrs85 led to the defective transport of proteins among the secreted proteins. Immunofluorescence analysis of TgM2AP and TgRON2 in TgTrs85-mAID-3HA parasites cultured in the presence or absence of IAA for 48 h. (H) Plaque assay measuring the growth of TgTrs85-mAID-3HA and parental strains in the presence or absence of IAA for 8 days. (I to L) Phenotypic characterization of TgTrs85-mAID-3HA mutants. Conditional ablation of TgTrs85 affected parasitic growth, attachment/invasion, replication, egression, and natural egress in cell culture. (M) Network representation of the TRAPP III complex interactome in T. gondii generated with Cytoscape software. The size of each point in the diagram indicates the size of the connectivity. The color of the lines from blue to red indicates the strength of coexpression from low to high. The thickness of the bars indicates the overall scores for the interaction between proteins. Heatmap analysis of differential lipids in TgTrs85-mAID-3HA (N) and 12HA-AID*-TgGS27 (O) strains in the presence or absence of IAA for 48 h. Download FIG S3, TIF file, 2.6 MB. Copyright © 2021 Cao et al. 2021 Cao et al. https://creativecommons.org/licenses/by/4.0/ This content is distributed under the terms of the Creative Commons Attribution 4.0 International license.

10.1128/mBio.01380-21.4 FIG S4 Generation of the 12HA-AID*-TgStx10, 6HA-AID*-TgStx12, and 12HA-AID*-TgVAMP4-2 strains and characterization of TgStx10, TgStx12, and TgVAMP4-2. (A, G, and L) Schematic representation of the AID*-based system used to inactivate TgStx10, TgStx12, and TgVAMP4-2 conditionally. (B, H, and M) Immunofluorescence analysis of TgStx10, TgStx12, and TgVAMP4-2 (green) in 12HA-AID*-TgStx10, 6HA-AID*-TgStx12, and 12HA-AID*-TgVAMP4-2 parasites cultured in the presence of 500 nM IAA or the vehicle (EtOH) for 16 h. (C, I, and N) Genomic PCR analysis using the indicated primers confirmed homologous recombination of the TgStx10, TgStx12, and TgVAMP4-2 genomic loci. (D, J, and O) Western blot analysis of 12HA-AID*-TgStx10, 6HA-AID*-TgStx12, and 12HA-AID*-TgVAMP4-2 parasites cultured in the presence of 500 nM IAA or vehicle (EtOH) for 0, 4, 8, and 16 h. (E and K) Conditional depletion of TgStx10 and TgStx12 led to defective vesicle transport of TgMIC2, TgM2AP, TgRON11, and TgROP4 in 12HA-AID*-TgStx10, and 6HA-AID*-TgStx12 parasites were cultured in the presence or absence of IAA for 24 h. (F and Q) Replication of 12HA-AID*-TgStx10 and 6HA-AID*-TgStx12 parasites after growth in the presence or absence of IAA for 24 h. (P) Red/green invasion assay examining the ability of 12HA-AID*-TgVAMP4-2 parasites to attach to and invade host cells in the presence or absence of IAA. The parasites were incubated for 4 h in the presence or absence of IAA before egression. (R). The egress of 12HA-AID*-TgVAMP4-2 parasites from host cells was determined by IFA. Parasites were grown in HFFs for 36 h in the presence or absence of IAA or the vehicle (EtOH) for 16 h before stimulation with A23187 for 5 min. Download FIG S4, TIF file, 2.3 MB. Copyright © 2021 Cao et al. 2021 Cao et al. https://creativecommons.org/licenses/by/4.0/ This content is distributed under the terms of the Creative Commons Attribution 4.0 International license.

Interestingly, our results also revealed an additional Qc SNARE (TgStx19), which lacks clear orthologues in humans and *Plasmodium* spp. and resided at a site similar to that of TgStx18. Therefore, it is reasonable to speculate that TgStx19 in T. gondii might have a function similar to the role of Use1 in mammalian cells. The depletion of TgStx18 and TgStx19 seemed to interfere with the inheritance of the apicoplast (Fig. 1C and F). However, the intracellular replication (Fig. 1D and G) and the lytic cycle of parasites (Fig. 1E and H) were severely interrupted as well when TgStx18 and TgStx19 were ablated. Upon treatment with IAA for 16 h, the parasites almost stopped growing and stayed in the single parasite stage, and few parasites achieved two parasites per vacuole. The very small amount of successfully divided parasites displayed severe morphological defects, which lost the banana shape and appeared spherical (Fig. 1C and F and Fig. S2B, F, and H). Furthermore, failure of apicoplast inheritance could be observed in some daughter parasites. Therefore, we concluded that TgStx18 and TgStx19 might participate in trafficking from the Golgi complex to ER and induced a widespread influence on parasite growth.

### A SNARE complex including TgGS27 is needed for protein trafficking at Golgi stacks.

**FIG 2:**
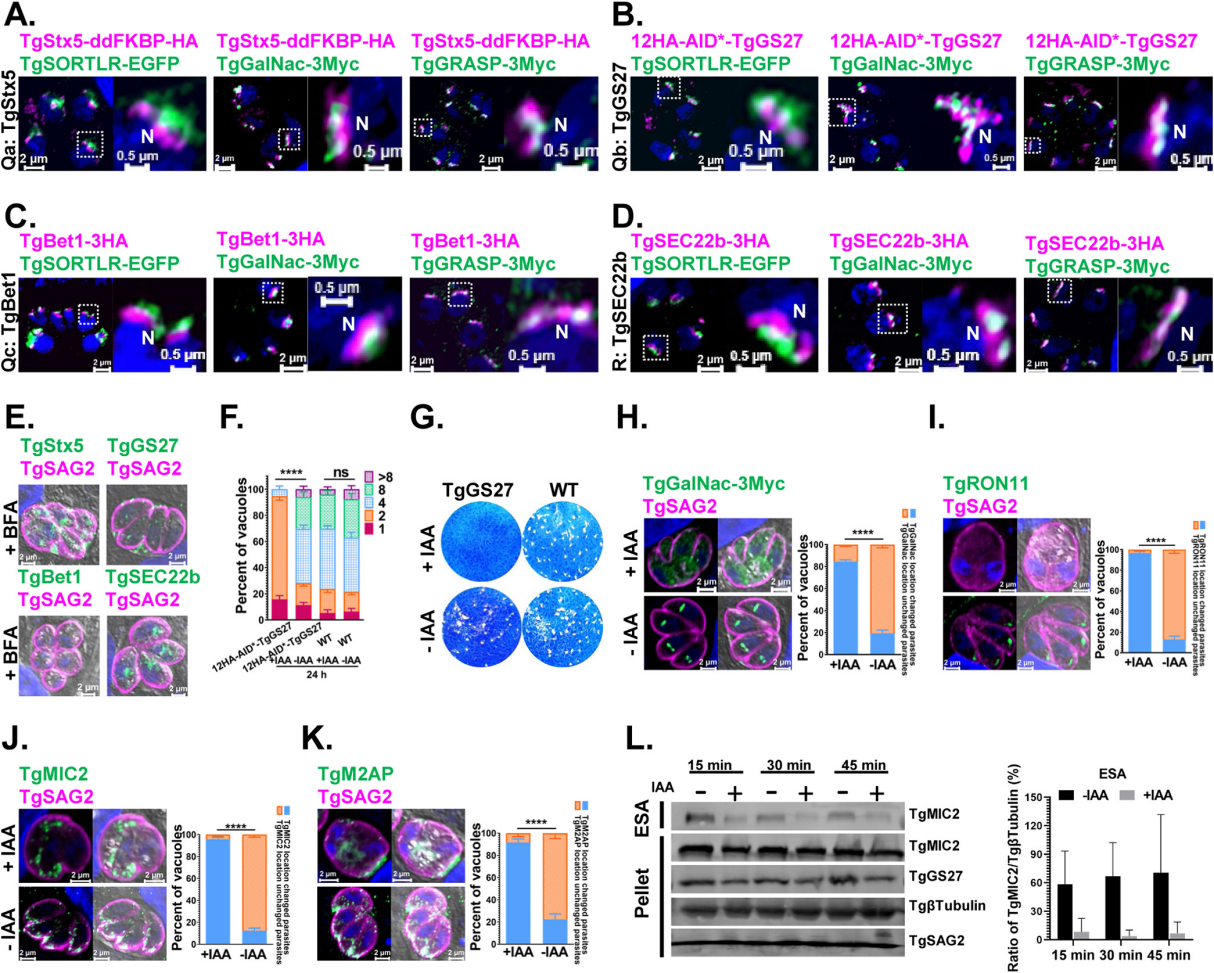
TgGS27 at the Golgi complex is required for parasite survival, and inactivation of TgGS27 led to the defective transport of proteins in the Golgi complex and secreted proteins. (A to D) Immunofluorescence analysis of the TgSTX5(Qa)/TgGS27(Qb)/TgBet1(Qc)/TgSec22b(R) SNAREs. TgSORTLR-EGFP, TgGalNac-3Myc, and TgGRASP-3Myc were used as *trans*-, *medial*-, and *cis*-Golgi markers, respectively. (E) BFA treatment impeded the localization of TgSTX5, TgGS27, TgBet1, and TgSec22b. (F) Replication of 12HA-AID*-TgGS27 parasites after growth in the presence or absence of IAA for 24 h. (G) Plaque assay measuring the growth of 12HA-AID*-TgGS27 and parental strains in the presence or absence of IAA. (H) Conditional depletion of TgGS27 caused TgGalNac to adopt a dispersed distribution within the cytosol of parasites. (I to K) Conditional depletion of TgGS27 led to the significant disruption of secreted proteins, including TgRON11, TgMIC2, and TgM2AP. Parasites were added to HFF cells to invade for 3 h under normal growth conditions and then grown in the absence or presence of IAA for 16 h. (L) Conditional depletion of TgGS27 affected secretion of the microneme protein TgMIC2 in ESAs and pellets, as determined by immunoblot analysis. The tachyzoites were incubated for 24 h in the presence or absence of IAA.

In mammalian cells, GS27 (Qb) is a component of ER-derived COPII transport vesicles and is required for anterograde trafficking to the ERGIC by forming a SNARE complex with Stx5 (Qa) Bet1 (Qc), and Sec22b (R). The GS27 also mediates retrograde trafficking from early and recycling endosomes to the Golgi stacks by complexing with Stx5 (Qa), Bet1 (Qc), and Ykt6 (R) (35). Conversely, the corresponding SNARE complex, including TgStx5, TgGS27, TgBet1, and TgSec22b, was detected primarily with the *cis*-Golgi marker TgGRASP (Fig. 2A to D), which indicates that ER-derived vesicles directly fuse with the *cis*-Golgi marker. The 12HA-AID*-tagged TgGS27 locus (Fig. S2J to M) showed a similar localization at Golgi stacks (Fig. 2D). We also observed that all four SNAREs were sensitive to Brefeldin A (BFA) treatment (Fig. 2E). TgGS27 is also required for the lytic cycle of the parasites (Fig. 2F and G). By staining TgGalNac, a medial Golgi protein, we found that intra-Golgi vesicle transport was disrupted in the TgGS27-deficient parasites (Fig. 2H). The transport of proteins destined for secretory organelles (TgMIC2, TgM2AP, and TgRON11) was investigated in parasites after TgGS27 was depleted. The signal of TgRON11 staining could not be seen when the parasites were treated with IAA for 24 h indicated that this protein could not accumulate in the organelle properly (Fig. 2I). However, it is hard to judge whether these proteins are properly delivered because of the severe morphological changes of TgGS27-deficient parasites (Fig. 2J and K). Instead, the secretion of TgMIC2 was further determined upon ethanol stimulation. The results indicated that the secretion of TgMIC2 was impaired when TgGS27 was depleted (Fig. 2L).

### The TRAPP III-specific subunit TgTrs85 is critical for intra-Golgi trafficking.

**FIG 3:**
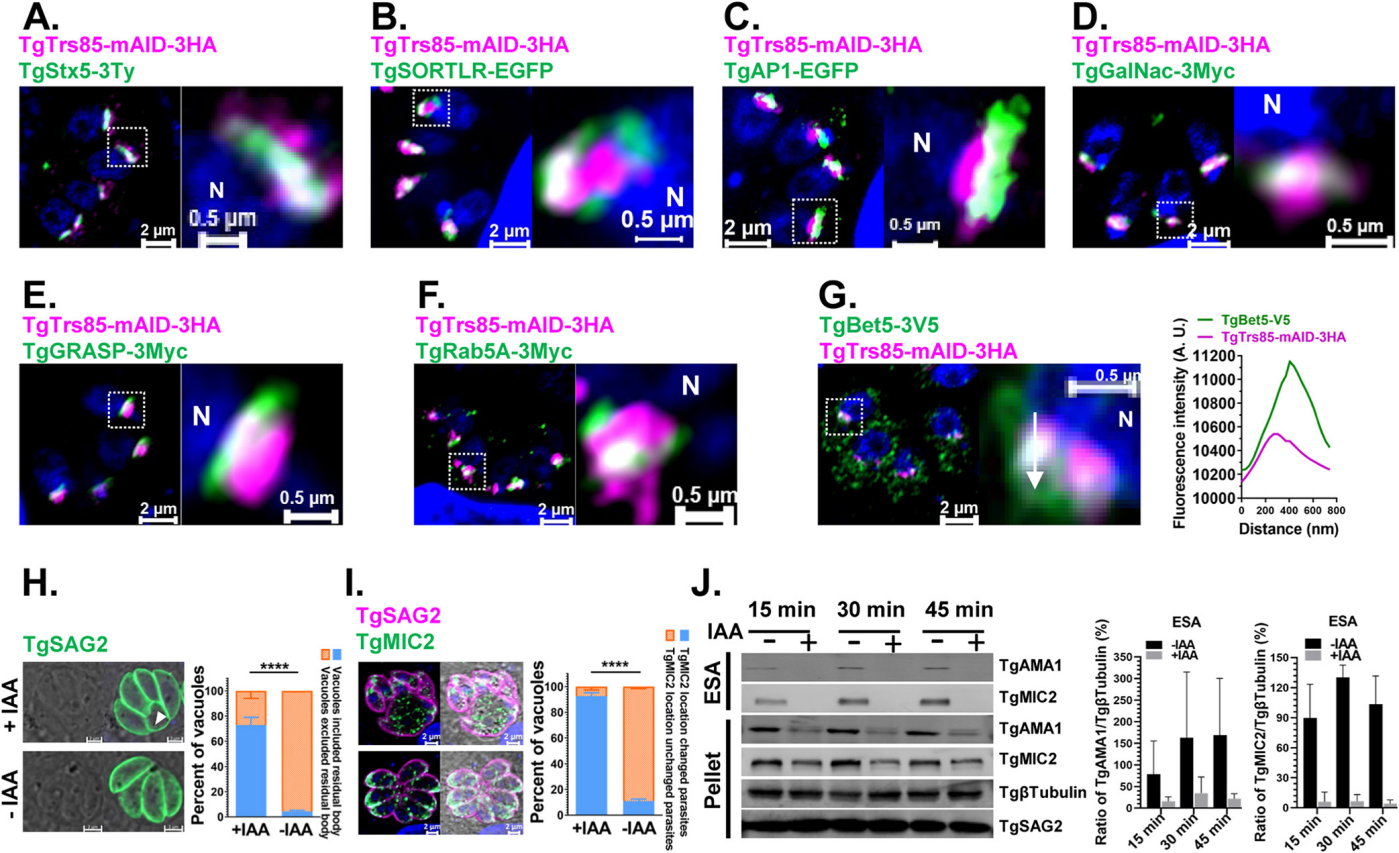
TRAPP III-specific subunit TgTrs85 colocalized with TgStx5 is necessary for the transport of secreted proteins at the Golgi. (A) Immunofluorescence analysis of TgStx5 colocalization with TgTrs85. (B to E) Immunofluorescence analysis of the TgTrs85-mAID-3HA strain expressing the *trans*-, *medial*-, and *cis*-Golgi markers TgSORTLR-EGFP, TgAPμ1-EGFP, TgGalNac-3Myc, and TgGRASP-3Myc. (F) Immunofluorescence analysis of TgRab5A partially colocalized with TgTrs85. (G) The TRAPP core component TgBet5 partially colocalized with TgTrs85. The colocalization of TgTrs85 is shown, and signal intensity plots across the TgBet5 distribution are graphed. (H) Arrow in the left panel indicates the accumulation of residual bodies. The right panel shows the quantification of PVs with residual bodies in IAA-treated cultures and control. (I) Depletion of TgTrs85 led to defective transport of the secreted proteins. The left panel reveals the immunofluorescence staining of TgMIC2 in TgTrs85-mAID-3HA parasites cultured in the presence or absence of IAA for 48 h. The right panel shows quantification of parasites with diffused staining of TgMIC2 in IAA-treated cultures and the control. (J) Immunoblot analysis of microneme secretion. The left panel reveals conditional depletion of TgTrs85 affected secretion of TgAMA1 and TgMIC2. The tachyzoites were incubated for 24 h in the presence or absence of IAA. The right panel shows the semiquantitative analyses of Western blot signals of TgAMA1 and TgMIC2 in the ESAs and pellet.

With the SNARE protein GS27, TRAPP III is needed for ER-Golgi transport in mammalian cells (36, 37). We endogenously tagged a putative TgTrs85, which is a TRAPP III-specific subunit, with mAID-3HA to analyze the localization of the complex (Fig. S3A to C). A clonal parasite line was isolated, and the localization of TgTrs85-mAID-3HA was examined by an immunofluorescence assay (IFA) using the Airyscan detector of a Zeiss LSM 880 or 980 instrument. Localization in the Golgi area was observed and confirmed by colocalization with the *trans*-, *medial*-, and *cis*-Golgi network markers TgStx5-3Ty, TgSORTLR, TgAPμ1, TgGalNac, TgGRASP, and TgRab5A (Fig. 3A to F). Overall, TgTrs85 accumulated close to the *medial*-Golgi marker and partially colocalized with ELC and *trans*- and *cis*-Golgi markers in the parasite. TgBet5, a subunit of the core heteroheptamer of TRAPP complexes, appeared at many sites within the parasites and partially colocalized with TgTrs85 (Fig. 3G), as indicated by white puncta (corresponding to the overlap of the two fluorophores). Similarly, parasite morphology was also disturbed when TgTrs85 was depleted by IAA treatment. However, in contrast with TgGS27 deficiency, morphological changes became apparent after 24 h of treatment, the phenotypes of TgTrs85 deficiency could only be seen clearly after 48 h of treatment (Fig. S3D to G) with IAA. Another difference is that a significant portion of the parasites carried enlarged residual bodies after 24 h of treatment (Fig. 3H). This phenomenon became more evident after 48 h of treatment (Fig. S3D to G). Again, similar to the TgGS27-depleted parasites, the secretion of microneme proteins was impaired (Fig. 3J), although it is difficult to judge whether these proteins were localized correctly (Fig. 3I and Fig. S3F and G). Further analysis proved that TgTrs85 was essential for the lytic cycle of T. gondii (Fig. S3H to L). As an approach to uncover functional information from genetic network analysis, a protein-protein interaction (PPI) network map of TRAPPIII in T. gondii was generated by using the retrieval of interacting genes (STRING) database (Fig. S3M) to screen all candidates that putatively interact with TRAPP III (TgTrs85, TgBet3, TgBet5, TgTrs20, TgTrs23, TgTrs31, and TgTrs33) (Fig. S3M and Data Set S1). We found that the repertoire of putative partners with known functions appeared to be highly reduced in T. gondii. SNAREs and small GTPases, including Rab1, TgStx5, TgGS27, TgSec22b, TgStx6, and TgStx18, were found in the interaction network.

10.1128/mBio.01380-21.9 DATA SET S1 Protein-protein interaction network of TRAPP III with TgTrs85. Download Data Set S1, XLSX file, 0.04 MB. Copyright © 2021 Cao et al. 2021 Cao et al. https://creativecommons.org/licenses/by/4.0/ This content is distributed under the terms of the Creative Commons Attribution 4.0 International license.

### The depletion of TgGS27 and TgTrs85 disrupted NEAT protein transport to the apicoplast.

**FIG 4:**
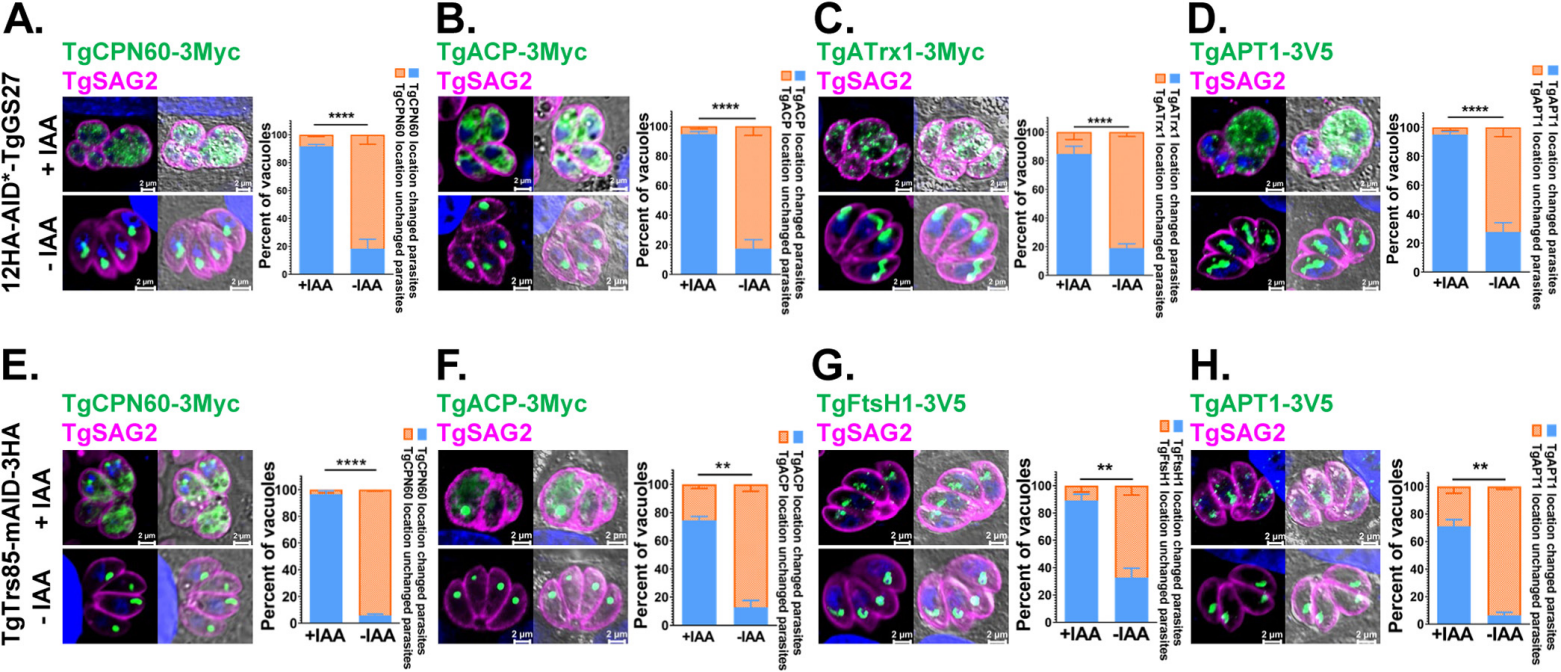
TgGS27 and TgTrs85 are required for the transport of NEAT proteins into the apicoplast. (A to D) Conditional ablation of TgGS27 affected the trafficking of TgCPN60-3Myc, TgACP-3Myc, TgATrx1-3Myc, and TgAPT1-3V5 to the apicoplast. (E to H) Conditional ablation of TgTrs85 affected the trafficking of two luminal proteins (TgCPN60-3Myc and TgACP-3Myc) and two outermost membrane proteins (TgAPT1 and TgFtsH1) to the apicoplast. Parasites were added to HFF cells, allowed to invade for 3 h under normal growth conditions, and then grown in the absence or presence of IAA for 16 h.

Previous reports have indicated that the Golgi pathway is involved in NEAT protein trafficking (38, 39). Therefore, we investigated the effect of TgTrs85 and TgGS27 depletion on apicoplast-targeted trafficking by examining the localization of TgCPN60-3Myc, TgACP-3Myc, TgFtsH1-3V5, and TgAPT1-3V5 or TgATrx1-3Myc. The conditional ablation of TgGS27 and TgTrs85 interfered with the entrance of TgCPN60, TgACP, TgAPT1, TgFtsH1, or TgATrx1 to the apicoplast after IAA treatment for 24 h (Fig. 4). When TgGS27 (Fig. 4A to D) and TgTrs85 (Fig. 4E to H) were depleted, NEAT proteins appear to be diffuse in the cytosol in most of the parasites (Fig. 4A, B, E and F), and multiple puncta could be observed in some parasite mutants (Fig. 4C, D, G, and H). The inheritance of the apicoplast in some daughter parasites was impaired (Fig. 4A and F).

### The ablation of TgGS27 and TgTrs85 disrupted the lipid metabolism of parasites.

**FIG 5:**
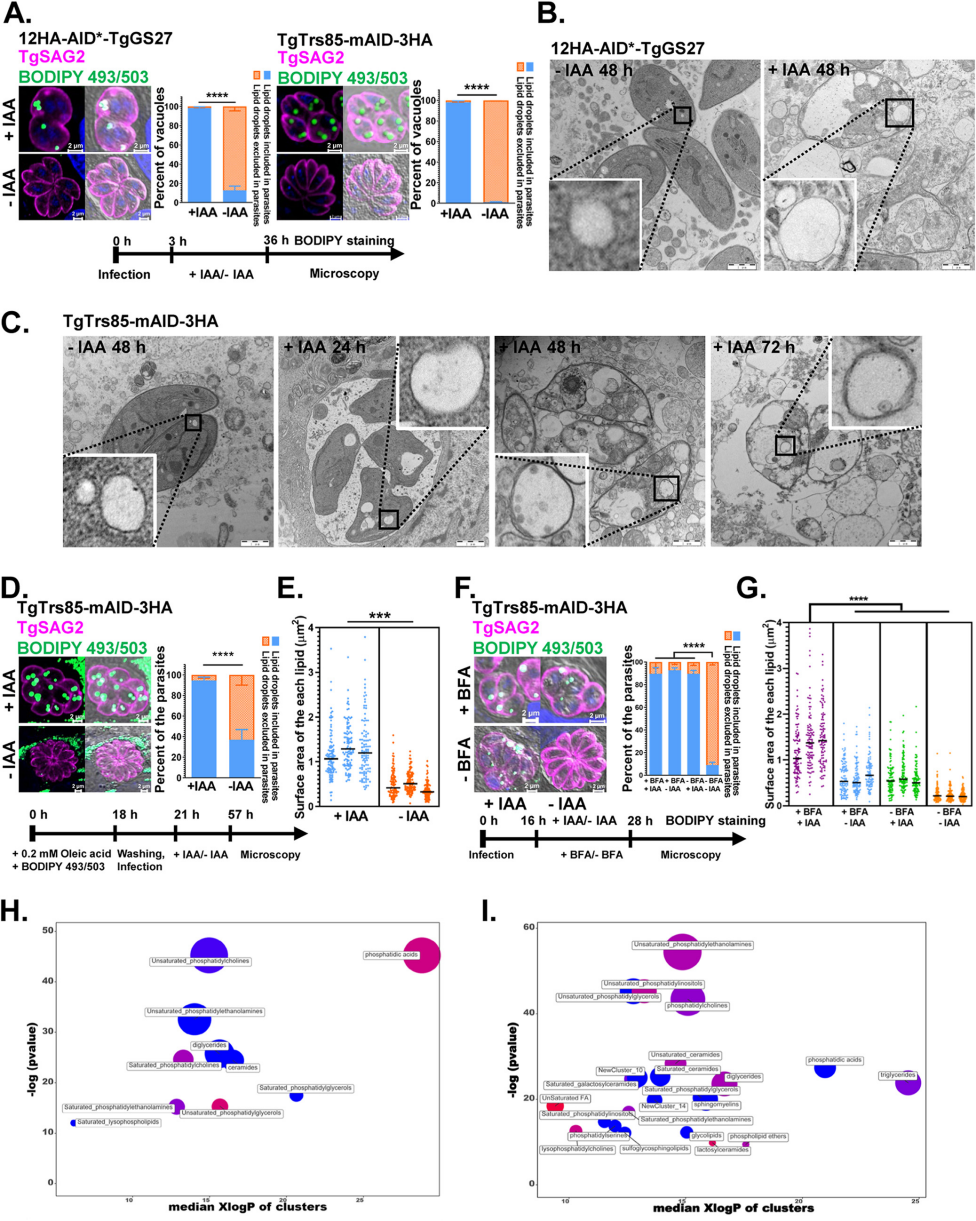
Inactivation of TgGS27 and TgTrs85 led to the accumulation of neutral lipid droplets (LDs) in the parasitic cytoplasm. (A) Conditional ablation of 12HA-AID*-TgGS27 and TgTrs85-mAID-3HA induced the accumulation of LDs in the parasitic cytoplasm. LDs labeled by BODIPY 493/503 contained in the cytoplasm of parasites were viewed by IFA. (B) A transmission electron micrograph of the 12HA-AID*-TgGS27 strain grown in the absence or presence of IAA for 48 h. (C) A transmission electron micrograph of the TgTrs85-mAID-3HA strain grown in the absence or presence of IAA for 24 h, 48 h, and 72 h. The LDs are shown in white. (D) TgTrs85 inactivation induced the accumulation of neutral lipids from the host cell in the parasites. (E) A significant difference in the surface area of LDs from the host was observed, and this difference was increased in parasites treated with IAA. (F) BFA and IAA treatments induced the additive accumulation of LDs in T. gondii. (G) Statistical analysis of the LDs in panel F. (H and I) Analysis of the lipid metabolites found in TgGS27- and TgTrs85-depleted parasites in the presence or absence of IAA. The color indicates the degree to which compound levels increased or decreased (red, increased; blue, decreased; pink, mixed) within each cluster. The parasites were cultured in the presence or absence of IAA for 48 h.

The Golgi complex is the primary location of lipid metabolism. Therefore, we assessed whether lipid metabolism would be disrupted if TgGS27 and TgTrs85 were abolished. The accumulation of neutral lipid droplets (LDs) was observed in the parasitic cytoplasm, as observed after the treatment of 12HA-AID*-TgGS27 and TgTrs85-mAID-3HA with IAA for 36 h and staining with BODIPY 493/503 (Fig. 5A). Electron microscopy micrographs also revealed the statistically significant accumulation of lipid bodies in the cytosol of TgTrs85- and TgGS27-deficient parasites (Fig. 5B and C). To investigate whether the accumulated LDs were from the host, exogenous oleic acid was added and stained with BODIPY 493/503 prior to parasite infection of human foreskin fibroblasts (HFFs). Fluorescence microscopy revealed that the overaccumulation of neutral lipids stained with BODIPY 493/503 was again present inside the cytoplasm of the parasites (Fig. 5D and E), which indicated that some of these lipids originated from the host cells. Previous studies suggested that the inhibition of vesicular trafficking in the ER and Golgi membrane by BFA also causes LD formation in microalgal species (40). We monitored the presence of LDs by staining the samples with a fluorescent indicator dye 3 h after parasite infection and treatment with or without IAA and BFA for 12 h. We found that the depletion of TgTrs85 and BFA treatment exerted a significant additive effect on the formation of LDs in the parasites, which indicated that the depletion of TgTrs85 affects additional lipid trafficking pathways in addition to its impact in interrupting Golgi trafficking (Fig. 5F and G). Specific sets of lipids in metabolomics can be defined by the relatedness of their chemical structures using chemical set enrichment statistics in chemical similarity enrichment analysis (ChemRICH) (41). To gain further insight into the disruption of lipid metabolism, the ChemRICH tool was used to detect the enrichment of metabolites in the TgTrs85- and TgGS27-deficient parasites. In total, 133 and 142 different lipids were screened and identified, respectively, after treatment with IAA for 48 h. Among these lipids, unsaturated phosphatidylethanolamines and phosphatidylglycerols were markedly decreased in parasites without TgTrs85 and TgGS27 (Fig. 5H and I and Fig. S3N and O and Data Set S2).

### TgStx10 and TgStx12 played important roles at the ELC.

**FIG 6:**
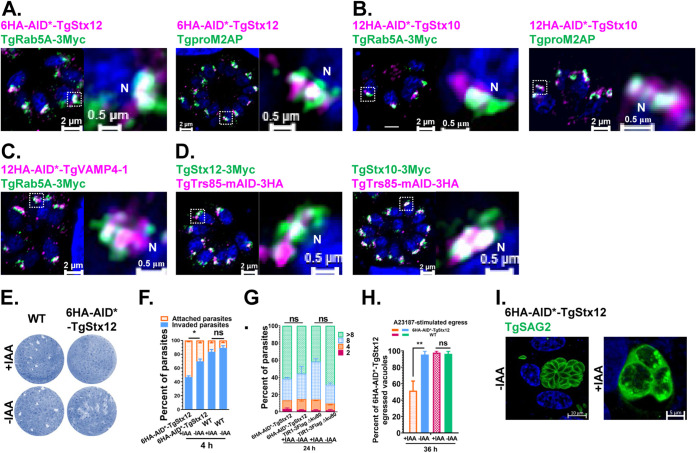
Characterizations of TgStx10 and TgStx12. (A to C) TgStx12, TgStx10, and TgVAMP4-1 localized at the ELC. (D) TgStx12 and TgStx10 partially colocalized with TgTrs85. (E) Plaque assay examining the growth of 6HA-AID*-TgStx12 parasites in confluent HFFs after 8 days in the absence or presence of IAA. (F) Red/green invasion assay examining the ability of 6HA-AID*-TgStx12 parasites to attach to and invade host cells. The parasites were incubated for 6 h in the presence or absence of IAA before egression. (G) Replication of 6HA-AID*-TgStx12 parasites after growth in the presence or absence of IAA for 24 h. (H) The egress of 6HA-AID*-TgStx12 parasites from host cells was determined by IFA. Parasites were grown in HFFs for 20 h under normal conditions, and then IAA or ethanol was added for 16 h before stimulation with A23187. (I) Conditional ablation of TgStx12 exhibits bright fluorescence stained by TgSAG2 in the lumen of the partial parasitophorous vacuole. The parasites were treated with auxin for 60 h.

Three SNAREs, TgStx10 (Qc), TgStx12 (Qa), and TgVAMP4-1 (R), were observed at the ELC and colocalized with TgRab5a and TgproM2AP (Fig. 6A to C). TgStx10-3Myc and TgStx12-3Myc also partially colocalized with TgTrs85-mAID-3HA (Fig. 6D). We observed that the depletion of TgStx12 affected the invasion and egress abilities of the parasites but had no effect on intracellular replication (Fig. 6E to H), which suggests a role in the maturation of MICs at this site. However, we found that the appropriate localization of MICs and RONs in the TgStx12-deficient parasites was not disturbed (Fig. S4K). When the parasites were treated with auxin for a longer time (60 h), severe morphological changes, including bright fluorescence stained by TgSAG2 in the lumen of the partial parasitophorous vacuole (PV), were observed (Fig. 6I). These data suggest additional roles of the TgStx12 complex at the ELC in addition to the transport of MICs.

### The depletion of TgVAMP4-2 blocked NEAT protein trafficking to the outermost membrane of the apicoplast.

**FIG 7:**
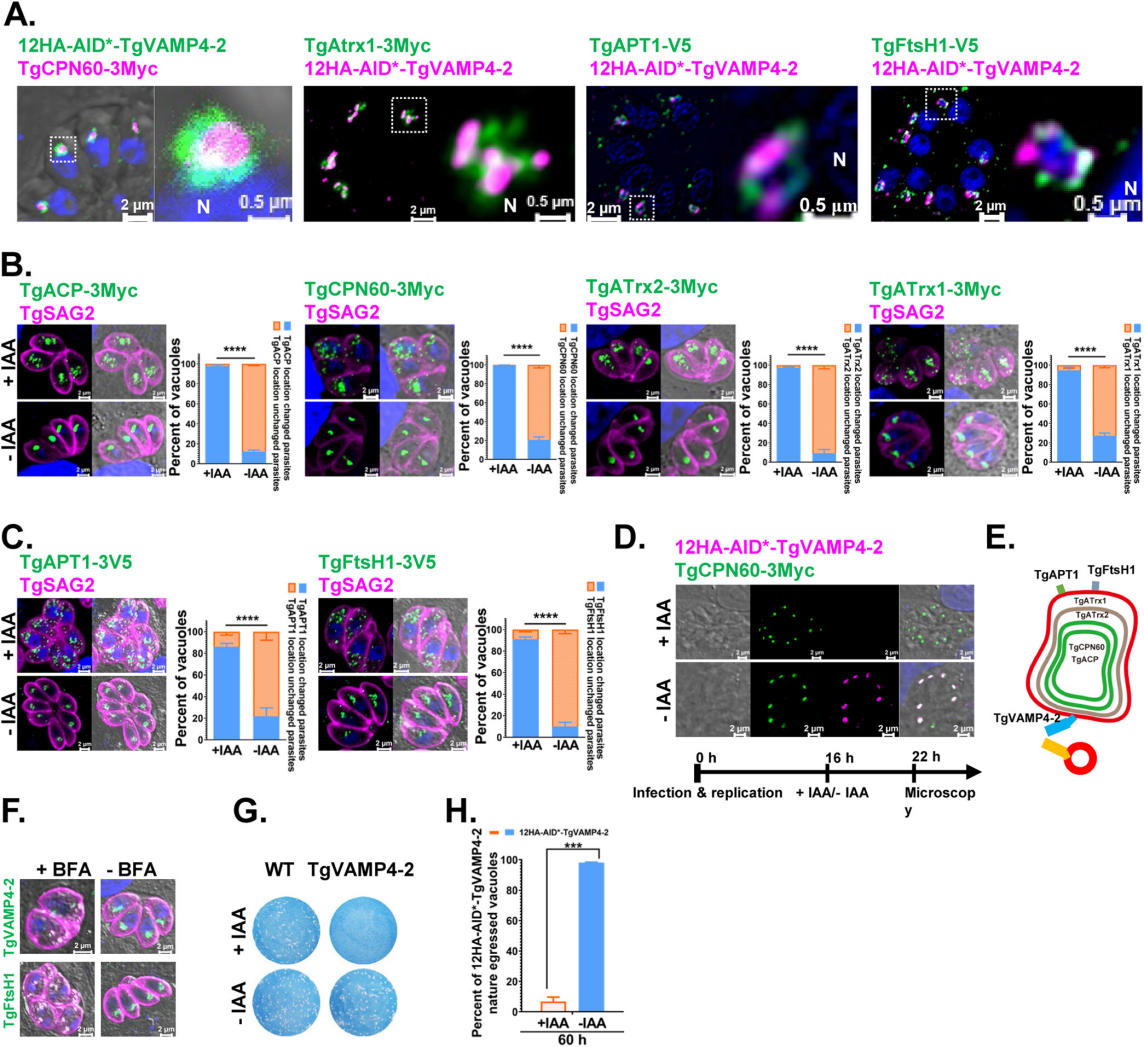
Outermost membrane R-SNARE of the apicoplast, TgVAMP4-2, plays a critical role in NEAT protein transport into the apicoplast. (A) Immunofluorescent investigation of targeting of the R-SNARE TgVAMP4-2 to the outermost membrane of the apicoplast compared to the locations of the apicoplast markers TgCPN60, TgAtrx1, TgAPT1, and TgFtsH1. (B) Conditional ablation of TgVAMP4-2 affected the trafficking of four luminal proteins (TgACP-3Myc, TgCPN60-3Myc, TgATrx2, and TgATrx1) to the apicoplast. (C) Conditional ablation of TgVAMP4-2 affected the trafficking of two outermost membrane proteins (TgAPT1 and TgFtsH1) to the apicoplast. Parasites were added to HFF cells, allowed to invade for 3 h under normal growth conditions, and then grown in the absence or presence of IAA for 16 h. (D) Apicoplasts were not destroyed by conditional ablation of TgVAMP4-2 after a short treatment. Shown is immunofluorescence analysis of the apicoplast protein TgCPN60 in 12HA-AID*-TgVAMP4-2 parasites cultured for 16 h and further treated with IAA for 6 h. (E) Schematic representation of the localization of the different NEAT markers associated with the apicoplast. (F) Treatment with BFA interferes with the localization of TgVAMP4-2 and TgFtsH1. (G) Plaque assay examining the growth of 12HA-AID*-TgVAMP4-2 parasites in confluent HFFs after 8 days in the absence or presence of IAA. (H) Parasites were grown and incubated for 60 h in the absence or presence of IAA before natural egression.

Strikingly, we observed an R-SNARE, TgVAMP4-2, at the apicoplast, which was confirmed by its colocalization with apicoplast lumen proteins (TgCPN60 and TgAtrx1) and membrane proteins (TgAPT1 and TgFtsH1). This SNARE appears to form a circle around the apicoplast similar to TgAPT1 and TgFtsH1. These data suggested that TgVAMP4-2 accumulated at the outermost membrane of the apicoplast (Fig. 7A).

We then examined the transport of TgCPN60, TgACP, TgATrx2, and TgATrx1 in TgVAMP4-2-depleted parasites by IFAT. To do this, we introduced a plasmid for the expression of 3Myc-tagged TgCPN60, TgACP, TgATrx2, or TgATrx1 into the 12HA-AID*-TgVAMP4-2 strain (Fig. S4L, M, N). The transport of all the protein markers was disrupted (Fig. 7B). Moreover, the outermost membrane markers (TgFtsH1 and TgAPT1), which were suggested to be transported through the Golgi pathway, were also affected upon TgVAMP4-2 depletion (Fig. 7C). We excluded the possibility that depletion of TgVAMP4-2 destroyed the entire apicoplast, as we observed NEAT protein trafficking. We cultured the parasites for 16 h and then treated them with IAA for an additional 6 h. Depletion of TgVAMP4-2 did not destroy the integrity of the organelle, since the TgCPN60 signal appeared normal (Fig. 7D and E). Our data revealed that the proper localization of TgVAMP4-2 itself is also dependent on Golgi trafficking, similar to the localization of TgFtsH1, as indicated by BFA treatment (Fig. 7F).

The viability of T. gondii without TgVAMP4-2 was affected, as indicated by plaque assays of HFFs (Fig. 7G). Still, the invasion efficiency of the parasites was not reduced after IAA treatment for 4 h, which indicated that TgVAMP4-2 is not involved in the invasion of host cells (Fig. S4P). The replication of TgVAMP4-2 was not affected by incubation in the presence of IAA for 24 h (Fig. S4O), which is similar to the delayed death phenotype often observed when apicoplast biogenesis is disrupted. The depletion of TgVAMP4-2 in the parasites affected their natural egress from host cells treated with IAA for 60 h but not their Ca^2+^-stimulated egress (Fig. 7H and Fig. S4R).

## DISCUSSION

Vesicular trafficking is involved in critical biological processes, including the secretory pathways responsible for transporting materials between different cell compartments. T. gondii has a highly polarized secretory system mediated via a singular Golgi apparatus (4). The transport of proteins destined for secreted organelles, exocytic vesicles, the inner membrane complex, and even the endosymbiotic apicoplast is associated with the secretory system in this parasite (42, 43). In the present study, we analyzed the functions of SNAREs, which are the key molecules involved in vesicular trafficking at the site of fusion between vesicles and the target membrane.

The most striking finding of this study is identifying an R-SNARE protein on the outermost membrane of the endosymbiotic apicoplast. Like the results obtained with other endosymbiotic organelles, apicoplast endosymbiosis results in the large-scale transfer of genes from the red algal endosymbiont to the host nucleus (44). A crucial step for successfully acquiring the apicoplast via secondary endosymbiosis is the development of mechanisms responsible for transporting nucleus-encoded apicoplast proteins back into the apicoplast (45). In this study, TgVAMP4-2 was found to be membrane bound and appeared to be located at the outermost apicoplast membrane in T. gondii. TgVAMP4-2 is the first SNARE protein found to be located on an endosymbiotic organelle. Detailed analysis indicated that TgVAMP4-2 governed the trafficking of four luminal proteins (TgACP-3Myc, TgCPN60-3Myc, TgATrx2-3Myc, and TgATrx1-3Myc) and two outermost membrane proteins (TgAPT1 and TgFtsH1) of the apicoplast. The biogenesis of the apicoplast in parasites was also impaired in 12HA-AID*-TgVAMP4-2 parasites treated with IAA. Our data suggest that TgVAMP4-2 is crucial for vesicular trafficking through the outermost membrane of the apicoplast. Therefore, the present study provides the first strong and solid evidence showing that the transport of NEAT proteins is mediated by a SNARE, which is thought to be involved in a vesicular trafficking pathway.

Our data also revealed that a Qa-SNARE (TgStx12), a Qc-SNARE (TgStx10), and an R-SNARE (TgVAMP4-1) reside at the ELC. The depletion of TgStx12 did not disturb the trafficking of MICs and RONs but affected the invasion and egress abilities of the parasites. Consistent with our study, a very recent report indicated that this phenomenon is associated with the processing activity of TgASP3 (15). In addition, the transport of MICs might not be the sole role of the TgStx12 complex at the Golgi stacks, because the absence of this protein for more extended periods substantially inhibited the viability of the parasites. Due to the possible role of ELC in endocytosis, it might be easy to understand that TgStx12 is also involved in this process.

TgStx18 localizes close to an ER marker but does not colocalize at the ERES with TgSec13, indicating that it probably resides at the ERAS, which is consistent with the finding of Stx18 in mammalian cells. Importantly, this study identified a Qc-SNARE, TgStx19, that might have a function similar to that of Use1 in mammalian cells. This SNARE does not share apparent homology with any of the SNAREs of humans, yeast, or *Plasmodium* species but showed similar localization to the conserved TgStx18 at the ER. The homology comparison results show that T. gondii lacks the tethering factor at ERAS, the Dsl1 complex (46). Therefore, a distinct Qc SNARE at this site might be necessary to adapt for a novel tethering complex that is waiting to be investigated.

Our results revealed that depletion of TgStx18 and TgStx19 seemed to affect the inheritance of the apicoplast. Nevertheless, it remained unclear whether the impairment of NEAT transport in parasite mutants was directly caused by a disturbance on the transporting route from the ER to the apicoplast or indirectly mediated by disruption of the entire secreting system. It will be interesting to test whether this distinctive SNARE plays a specific role in the vesicular trafficking pathway in T. gondii (for example, the trafficking route of NEAT proteins to the apicoplast). This idea should be investigated due to the lack of a homolog in *Plasmodium* parasites, and no clear orthologue has been found for TgVAMP4-2. From an evolutionary view, the diversity of SNAREs on the outermost membrane of apicoplasts and the ER suggests that a similar endosymbiotic system of *Apicomplexa* evolved independently, even though cryptic plastids are typical of different parasites. This idea is supported by results from a single-cell analysis that indicated that endosymbiotic organelles evolved independently at least three times (47).

Previous studies have suggested that the Golgi pathway is involved in NEAT protein transport to the apicoplast (39). Our results provide further evidence supporting this model. Conditional ablation of the Golgi-localized SNARE TgGS27 led to a diffuse distribution of TgGRASP (*cis*-Golgi), which indicates that it functions in ER-Golgi trafficking in T. gondii. Consequently, the trafficking of proteins destined for secretory organelles, exocytosis, and the IMC complex was disturbed in the absence of TgGS27. We also provide clear evidence that an MTC, TRAPPP III, predominantly localizes at central Golgi membrane in T. gondii and plays a critical role in the secretion system. However, the severe morphological changes in the mutant parasites make it difficult to judge whether the proteins destined to the secretory organelles were delivered correctly. Therefore, further careful analysis is needed to analyze how the exocytosis of secretory organelles was disturbed. The transport of NEAT proteins to the endosymbiotic apicoplast was indeed affected in TgGS27- and TgTrs85-depleted parasites.

In addition, according to our data and the known functions of their orthologues in other organisms, TgGS27 and TgTrs85 should both mediate ER-Golgi trafficking in T. gondii. However, we noticed TgTrs85 localized at a broader range of sites than TgGS27, and some phenotypes, such as enlarged residual bodies observed in the TgTrs85-deficient parasites, were not found in the TgGS27-depleted parasites. These results indicated that further investigation of the molecular mechanisms that they are involved in is still necessary. In addition, our analysis revealed that another Golgi-residing Qb, SNARE TgGS28, seems to be conserved. Therefore, it would be interesting to investigate its function in the early secretory system of T. gondii.

Lipid metabolism in apicomplexans is essential for the process of intracellular development, the generation of infectious progeny parasites, and long-term persistence in the host cell (48, 49). Apicomplexan parasites obtain some lipids through host cell scavenging and combine this process with *de novo* synthesis to maintain parasite propagation and survival (8). However, the maturation process and the trafficking mechanism of lipid droplets in T. gondii are still unclear. In this study, the accumulation of host-derived neutral lipids in the cytoplasm of parasites and disturbance of lipid metabolism was observed in TgGS27- and TgTrs85-deficient T. gondii by transmission electron microscopy (TEM) and IFA. Added to the fact that BFA treatment had an additive effect on lipid accumulation, this data depletion of TgTrs85 affects additional lipid metabolic or trafficking pathways in addition to its impact in interrupting Golgi trafficking. To our knowledge, this study provides the first mention of the TRAPP III complex in regulating the biogenesis of LDs. TRAP III acts as a GEF and activates Rab1 in the secretory pathway (31), and TRAPP II activates Rab18 to regulate the LD size on the surface (50). Whether TgTrs85 has a similar function to activate Rab GTPase and the detailed mechanism needs further investigation in future studies.

In summary, our investigation of SNAREs revealed the critical molecular mechanisms of vesicular trafficking to secretory organelles and the apicoplast. These results provide insights into the mechanisms involving the vesicular trafficking network in T. gondii.

## MATERIALS AND METHODS

### Cultivation of parasite strains and host cells.

T. gondii RH, RHΔhxgprt, and RHΔhxgprtΔku80TIR1-flag were cultivated in human foreskin fibroblasts (HFFs) or Vero cells.

### Construction of T. gondii expression plasmids.

The CRISPR/Cas9 system was used in this study as described previously (51). The pCD-Cas9 vector harbored the Cas9 gene and the ToxoU6 promoter to drive guide RNA (gRNA). The target sequences of TgTrs85 (ToxoDB, TGGT1_214610), TgGS27 (ToxoDB, TGGT1_271060), TgStx10 (ToxoDB, TGGT1_300290), TgVtila-1 (ToxoDB, TGGT1_242080), TgVAMP4-1 (ToxoDB, TGGT1_248100), TgStx18 (ToxoDB, TGGT1_267530), TgStx19 (ToxoDB, TGGT1_208010), and TgVAMP4-2 (TGGT1_246610) with the gRNA scaffold were amplified and inserted into pU6-Cas9 at the PmeI site (Fig. S2 to S4). Fragments of TgSORTLR-EGFP, TgAPμ1-EGFP, TgGalNac-3Myc, TgGRASP-3Myc, TgRab5A-3Myc, TgBet5-3V5, TgStx5-ddFKBP-3HA, TgStx5-3Ty, 3HA-TgBet1, 3HA-TgSEC22b, TgCPN60-3Myc, TgACP-3Myc, TgATrx2-3Myc, TgATrx1-3Myc, TgFtsH1-3V5, TgAPT1-3V5, TgSAG1-GFP-HDEL-3Myc, TgERD2-3Myc, and TgSEC13-3V5 were introduced into the pBluescript-DHFR vector under the control of the promoter of TgGRA1 or Tgβtubulin.

### Generation of transgenic T. gondii strains.

For the genomic tagging of endogenous TgTrs85 with mAID-3HA at the C terminus, the T. gondii RHΔhxgprtΔku80TIR1-flag strain was transfected with this fragment and pCD-Cas9-TgTrs85 to add an mAID-3HA tag and then screened for locus insertion by PCR. In contrast, for tagging the endogenous genomic loci of TgGS27, TgStx10, TgStx12, TgStx18, TgStx19, TgVAMP4-1, and TgVAMP4-2 with 12HA-AID* at the N terminus, the tag was amplified with 40-bp sequences upstream and downstream of the start codon. The RHΔhxgprtΔku80TIR1-flag strain was transfected with these fragments and pCD-Cas9-TgGS27, TgStx10, TgStx12, TgStx18, TgStx19, and TgVAMP4-2 to insert a 12HA-AID* tag and screened for locus insertion by PCR. The accuracy of the integration was assessed by genomic PCR analysis and IFA (Fig. S2 to S5).

To express the marker proteins, parasites were generated by electroporation of 6 × 10^6^ tachyzoites with 60 μg of TgSORTLR-EGFP, TgAPμ1-EGFP, TgGalNac-3Myc, TgGRASP-3Myc, TgRab5A-3Myc, TgBet5-3V5, TgStx5-ddFKBP-3HA, TgStx5-3Ty, TgBet1-3HA, TgSEC22b-3HA, TgCPN60-3Myc, TgACP-3Myc, TgATrx2-3Myc, TgATrx1-3Myc, TgFtsH1-3V5, or TgAPT1-3V5 plasmid. The next day, the transfected parasites were selected with pyrimethamine over three passages. Although the Dsl1 complex plays a major role in Golgi-to-ER retrograde transport located at ERAS in eukaryotes, none of the complex subunits are found in T. gondii (24, 34). For the localization of ERAS in T. gondii, endoplasmic reticulum deficient-2 (TgERD2, TGGT1_256000) (52), TgSAG1-GFP-HDEL-3Myc (3), and TgSec13 (ER exit site marker, TGGT1_201700) (34) were used. TgERD2, TgSAG1, and TgSec13 were amplified from T. gondii cDNA and subcloned into plasmid pBluescript-DHFR under the control of the T. gondii TgGRA1 or Tgβtubulin promoter and fusing a 3× Myc, EGFP-HDEL, or 3× V5 tag to the 3′ end, respectively.

10.1128/mBio.01380-21.5 TEXT S1 Supplementary experimental procedures. Download Text S1, DOCX file, 0.03 MB. Copyright © 2021 Cao et al. 2021 Cao et al. https://creativecommons.org/licenses/by/4.0/ This content is distributed under the terms of the Creative Commons Attribution 4.0 International license.

10.1128/mBio.01380-21.6 TABLE S1 List of T. gondii SNARE proteins and TRAPP complex subunits. Download Table S1, DOCX file, 0.02 MB. Copyright © 2021 Cao et al. 2021 Cao et al. https://creativecommons.org/licenses/by/4.0/ This content is distributed under the terms of the Creative Commons Attribution 4.0 International license.

10.1128/mBio.01380-21.7 TABLE S2 Antibodies/antisera used throughout this study are indicated. Download Table S2, DOCX file, 0.02 MB. Copyright © 2021 Cao et al. 2021 Cao et al. https://creativecommons.org/licenses/by/4.0/ This content is distributed under the terms of the Creative Commons Attribution 4.0 International license.

10.1128/mBio.01380-21.8 TABLE S3 Primer sequences used for amplification and sequencing. Download Table S3, DOCX file, 0.02 MB. Copyright © 2021 Cao et al. 2021 Cao et al. https://creativecommons.org/licenses/by/4.0/ This content is distributed under the terms of the Creative Commons Attribution 4.0 International license.

10.1128/mBio.01380-21.10 DATA SET S2 Metabolic differentiators and chemical similarity enrichment analysis of the metabolites in 12HA-AID*-TgGS27 and TgTrs85-mAID-3HA strains in the presence or absence of IAA for 48 h. Download Data Set S2, XLSX file, 0.1 MB. Copyright © 2021 Cao et al. 2021 Cao et al. https://creativecommons.org/licenses/by/4.0/ This content is distributed under the terms of the Creative Commons Attribution 4.0 International license.
