# Supplementary figures and images for: The Dissection of SNAREs Reveals Key Factors for Vesicular Trafficking to the Endosome-like Compartment and Apicoplast via the Secretory System in Toxoplasma gondii
Source: mBio. 2021 Aug 3;12(4):e01380-21. doi: 10.1128/mBio.01380-21 (PMC8406237; doi:10.1128/mBio.01380-21)

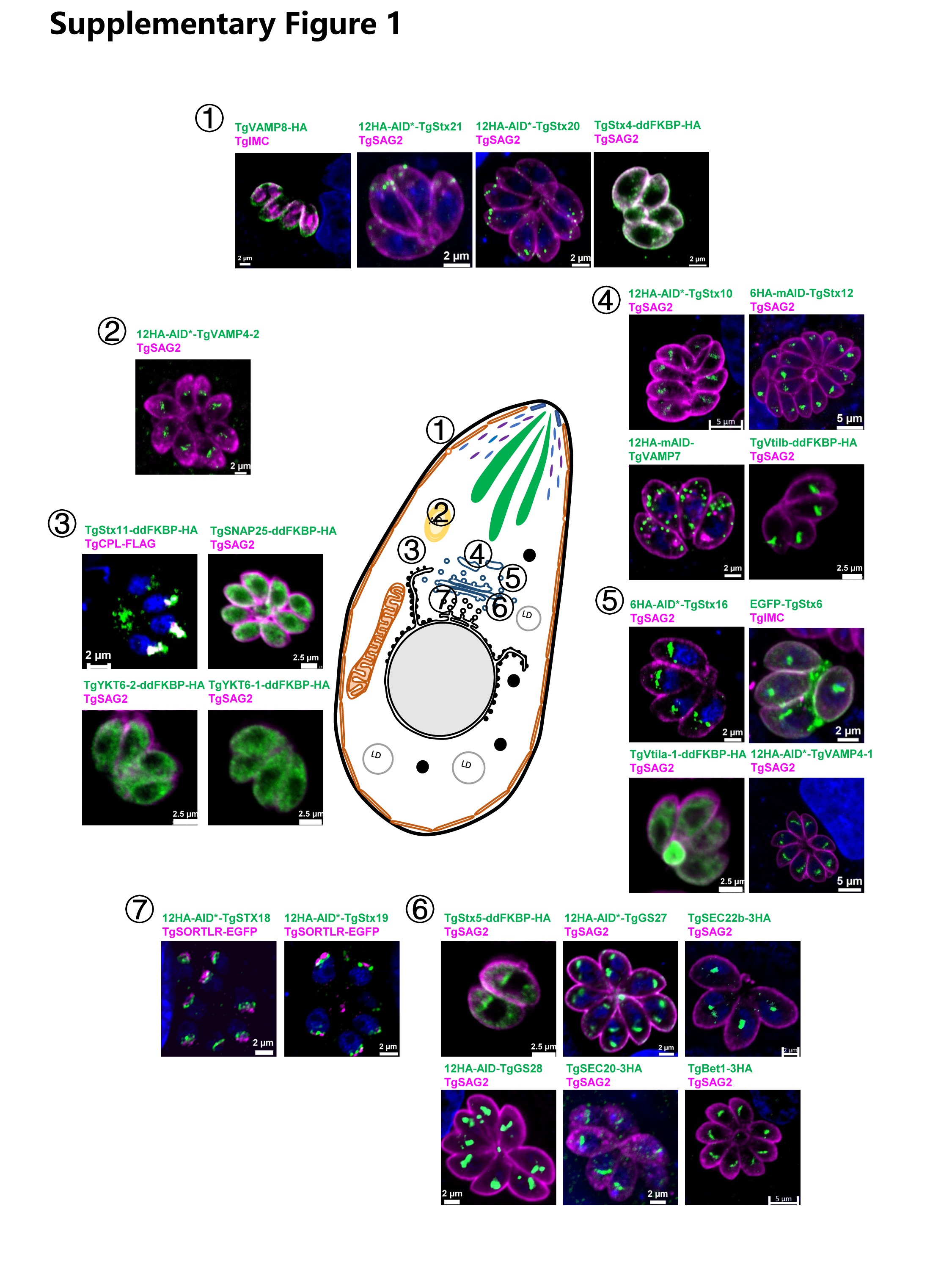

Supplement: FIG S1 [file mbio.01380-21-sf001.tif]

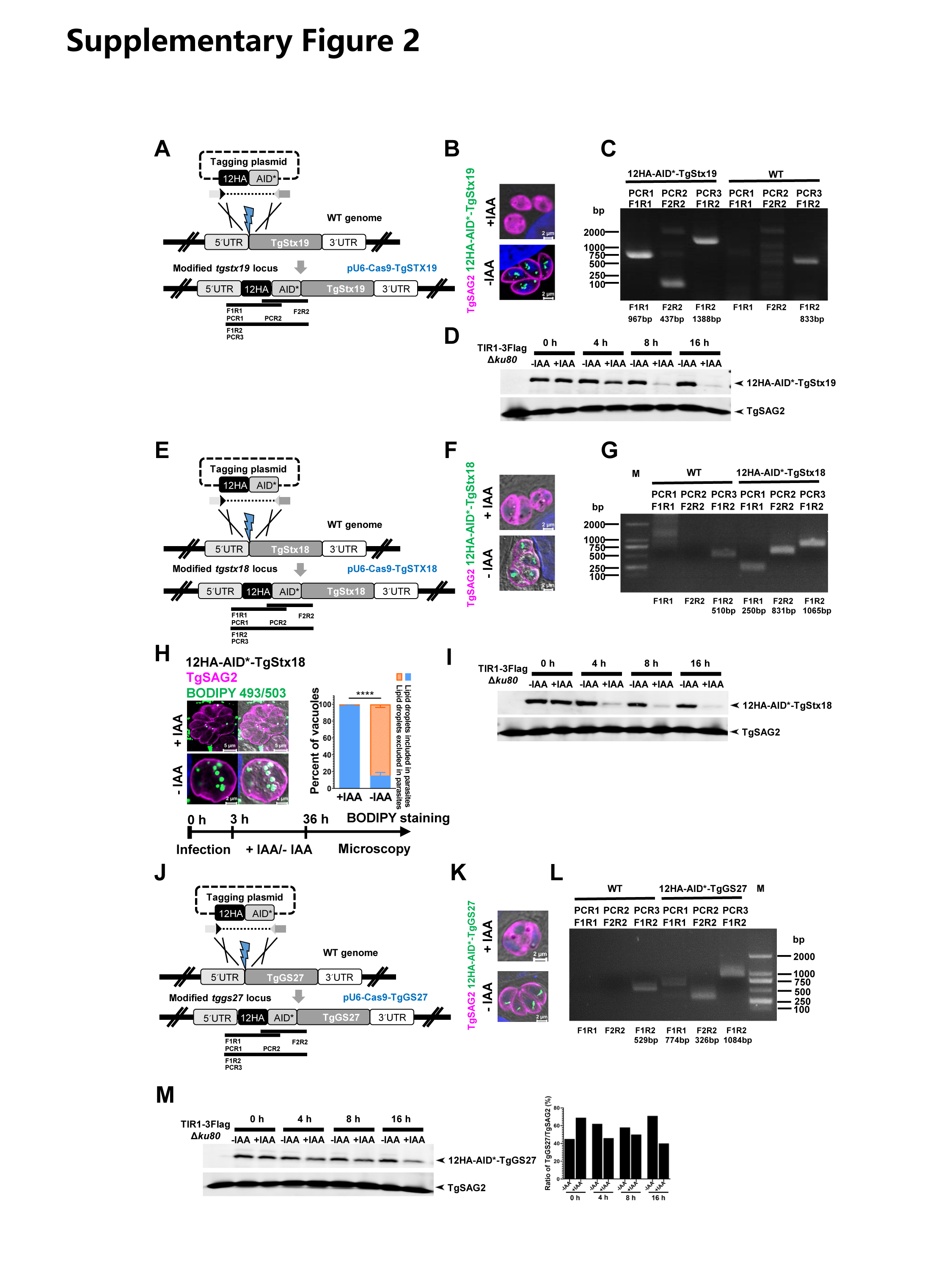

Supplement: FIG S2 [file mbio.01380-21-sf002.tif]

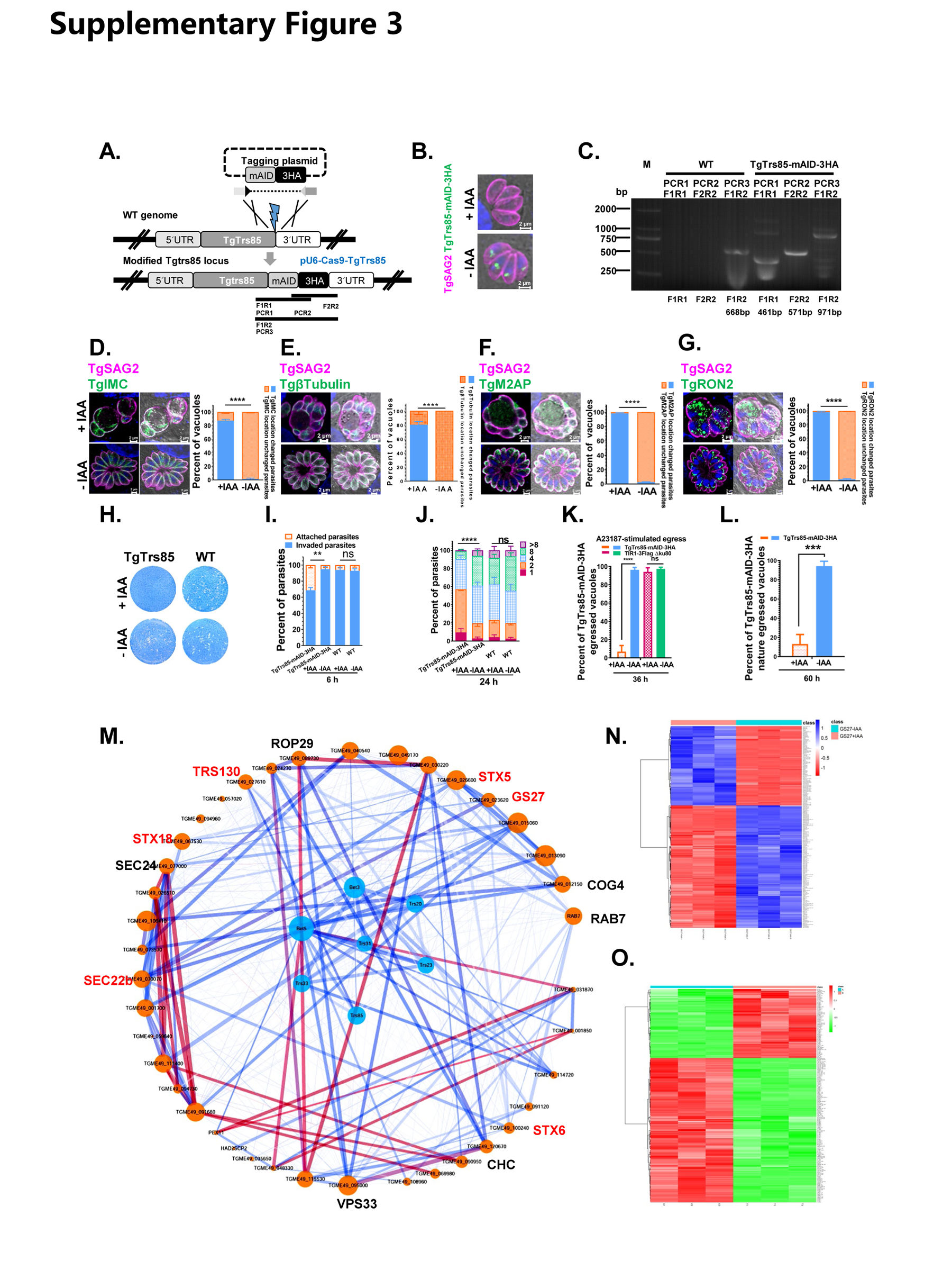

Supplement: FIG S3 [file mbio.01380-21-sf003.tif]

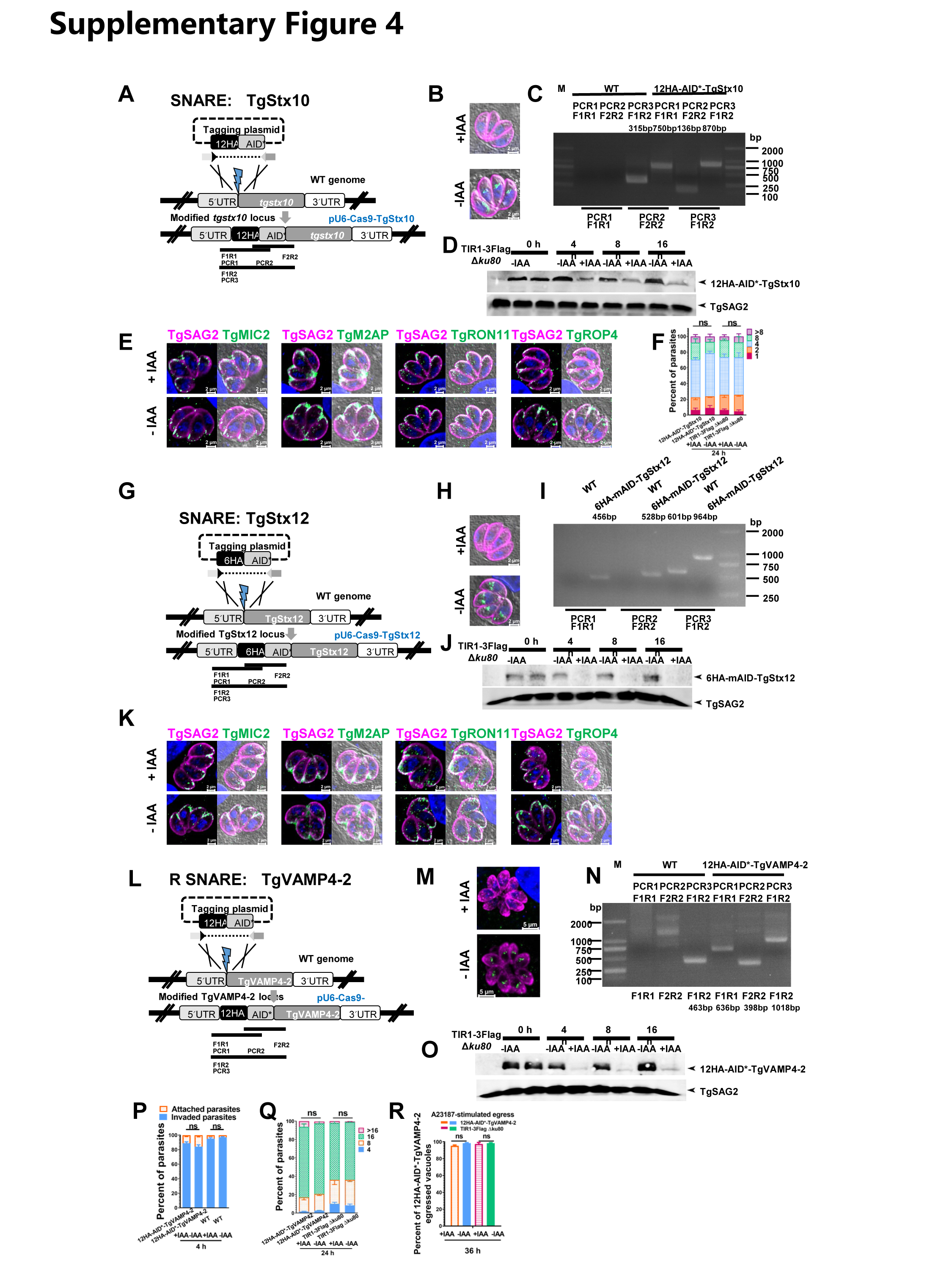

Supplement: FIG S4 [file mbio.01380-21-sf004.tif]
