# Supplementary material for: The Dissection of SNAREs Reveals Key Factors for Vesicular Trafficking to the Endosome-like Compartment and Apicoplast via the Secretory System in Toxoplasma gondii
Source: mBio. 2021 Aug 3;12(4):e01380-21. doi: 10.1128/mBio.01380-21 (PMC8406237; doi:10.1128/mBio.01380-21)
Supplement: TEXT S1 [file mbio.01380-21-t0001.docx]

**Supplementary Experimental Procedures**

**Bioinformatics analysis of TRAPP complex subunits.** Human, Arabidopsis, and yeast TRAPP complex subunit sequences were retrieved from the Saccharomyces Genome Database or GenBank. BLASTp was applied to identify TRAPP subunit homologues of *T. gondii* in the *Toxoplasma* genomics resource (ToxoDB) (S1 Table). TRAPP domains from conformant sequences were scanned using the NCBI Conserved Domain Database (CDD). To predict the associated interactors of the predicted TRAPP III complex, the search tool for the retrieval of interacting genes (STRING) (https://string-db.org) database was applied to predict functional interactions of proteins, including both known and predicted protein-protein interactions (PPIs). An active interaction source of coexpression limited to *T. gondii* was applied to construct the PPI networks. Cytoscape software version 3.8.2 (https://cytoscape.org/) was used to visualize the PPI network.

**IFA and confocal microscopy.** The intracellular parasites were fixed in 3% formaldehyde for 30 min at 4°C, permeabilized with 0.3% (v/v) Triton X-100 for 30 min, and blocked in PBS containing 5% BSA. The cells were incubated for 1 h with primary antibodies. The secondary antibodies conjugated to Alexa Fluor 488 or Alexa Fluor 594 (Invitrogen) were used at 1:1000 dilution. The fluorescence was detected using an LSM 980 laser-scanning confocal microscope (Zeiss) equipped with a 63×/1.4 or 100×/1.4 oil objective. The mean values and standard deviations are calculated from three independent experiments. Antibodies used in this study are listed in Supplementary Table 2.

**Immunoblot analyses and electron microscopy.** Freshly released tachyzoites or other samples were boiled in 4×SDS-PAGE sample buffer (Solarbio, S1052) and separated on 10% polyacrylamide gels. Proteins were transferred to nitrocellulose membranes. Each membrane was blocked with PBS containing 5% skim milk for 60 min at room temperature and then incubated with primary antibodies (1:1000). Each membrane was washed three times with PBST and then incubated with Alexa Fluor 680- and 800-conjugated goat anti-mouse or anti-rabbit secondary antibodies (1:5000) in 5% skim milk. Rabbit anti-TgSAG2 antibodies were used as loading controls. The PVDF membranes were scanned using an LI-COR Odyssey infrared imaging system at an intensity = 5 at wavelengths of 700 and 800 nm. The data shown are mean values ± SD (error bar) from three independent experiments.

For transmission electron microscopy (TEM), infected monolayers were fixed with 2.5% glutaraldehyde in 0.1 M phosphate buffer, post-fixed in osmium tetroxide, dehydrated in acetone, and embedded in 812 Epon resin. The sections were embedded with uranyl acetate followed by lead citrate, and the stained sections were viewed with a Hitachi H-7650 transmission electron microscope.

**Brefeldin A treatment.** A brefeldin A (BFA) solution was prepared in ethanol to a concentration of 10 mg/ml and stored at –30°C. The TgTrs85-mAID-3HA, TgSTX5-ddFKBP-HA, 12HA-AID*-TgGS27, 3HA-TgBet1 and 3HA-TgSEC22b strains were incubated with fresh medium containing 1 μg/ml BFA at 37°C for 4 h.

**Quantitative analyses of neutral lipid droplet sizes in parasites.** The numbers of neutral lipid droplet in the cytoplasm of parasites of the TgTrs85-mAID-3HA, 12HA-AID*-TgGS27, and 12HA-AID*-TgStx18 strains were determined by staining with BODIPY 493/503 in the absence and presence of IAA. The LD areas were calculated using ImageJ software. The data shown are representative of results from three independent replicates.

**Assays for assessing the uptake of neutral lipid fluorescent dye from host cells.** HFF cells were pretreated with 0.3 mM oleic acid and 50 μM BODIPY 493/503 for 18 h, thoroughly washed with PBS, and then chased for 3 h in DMEM. The neutral lipids of HFF cells were pre-loaded by BODIPY staining. The cells were then infected with the TgTrs85-mAID-3HA strain at 36 h before fixation for immunostaining. For quantification of neutral lipid uptake from host cells by the TgTrs85-mAID-3HA strains, fluorescence images were acquired with a Zeiss LSM 980 laser-scanning confocal microscope. The fields were randomly chosen, and the numbers of BODIPY-stained LDs within the parasites were quantified. The data shown are representative of results from three independent replicates. The individual LD area was calculated by ImageJ.

**Plaque assay.** Plaque assays were performed using a six-well plate containing monolayers of HFF cells infected with 100 parasites per well in the absence or presence of IAA. The cells were fixed at 8 days post-infection in 100% cold methanol for 45 min, and stained by Giemsa for 40 min. Images were processed using Adobe Photoshop (version 11.0.1).

**Red/green invasion assays.** For the analysis of the parasites’ ability to invade Vero cells, a total of 0.5×10^6^ extracellular parasites were incubated for 6 h in the presence or absence of IAA at natural egress condition. The freshly egressed *T. gondii* were allowed to invade Vero cells for 4 h. IFA was performed using a mouse anti-TgSAG2 antibody (1:2000) prior to permeabilization to stain extracellular parasites. Parasite-infected cells were then permeabilized with 0.3% Triton X-100 prepared in PBS for 15 min at room temperature. All parasites were then labeled by a rabbit anti-TgSAG2 antibody (1:2000). The number of invaded parasites was calculated by subtracting the number of extracellular parasites (co-labeled) from the total number of parasites in randomly selected fields. The data shown are representative of results from three independent replicates.

**Replication assay.** To evaluate the replicative ability of knockout parasites, Vero cells in six-well plates were infected with 5×10^5^ freshly released tachyzoites. After 2 h of invasion under normal growth conditions, the uninfected parasites were washed away with PBS. The infected parasites were cultured for another 24 h in presence or absence of IAA for 24 h. Cells were fixed with 4% paraformaldehyde and then permeabilized with 0.3% Triton X-100 prepared in PBS. The parasites were then detected by IFA using an anti-TgSAG2 antibody and visualized with Alexa Fluor 488-conjugated goat anti-rabbit IgG. The number of parasites per vacuole was determined, with 100 vacuoles examined per condition. The mean values and standard deviations are calculated from three independent experiments.

**Induced egress assay.** Freshly lysed egressed parasites were collected, counted, and allowed to infect and growth in 12-well plates (10^5^ parasites per well) under the normal condition for 20 h. After that, we added the IAA or vehicle for an additional 16 h treatments before starting the simulation. After a total of 36 h, the wells were washed three times by PBS before chemical induction. Intracellular PVs were treated with Hank’s balanced salt solution containing 3 µM A23187 (Sigma), a Ca^2+^ ionophore. The parasites were then fixed and permeabilized. IFA was performed with rabbit anti-TgSAG2 and mouse anti-GRA7 polyclonal antibodies to label the parasite plasma membrane and PV membrane, respectively. The percentage of vacuoles that displayed egress upon A23187 treatment was determined by examining 200 vacuoles under a microscope. Mean values from three independent experiments are shown.

**Natural egress assay.** Freshly lysed egressed parasites were collected, counted, and allowed to infect and growth in 12-well plates (10^5^ parasites per well) under the normal condition for 20 h. We then added the IAA or vehicle for an additional 40 h treatments. The percentage of 200 egressed vacuoles was analysed after 60 h.p.i. without the treatment of the Ca^2+^ ionophore. IFA was performed as same as above. The data shown are mean values ± SD (error bar) from three independent experiments.

**Microneme secretion assay.** A total of 2×10^8^ extracellular tachyzoites were incubated for 24 h in the presence or absence of IAA before egression and pelleted by centrifugation at 1000 × g for 10 min at room temperature. Samples were washed in incubated endobuffer (20mM Tris-H_2_SO_4_, 106 mM sucrose, 44.7 mM K_2_SO_4_, 10 mM MgSO_4_, 5 mM glucose, 3.5 mg/ml BSA, pH 8.2), resuspended in 100 µl of serum-free media containing 2% ethanol, incubated for 15 min, 30 min, and 45 min at 37°C, and then pelleted by centrifugation at 1000 × g for 10 min at 4°C. The supernatant containing excreted-secreted antigens (ESAs) was collected by centrifugation at 2000 × g for 15 min at 4°C. The pellet was resuspended in 4 × SDS-PAGE loading buffer and boiled for electrophoresis and immunoblotting. Mean values from three independent experiments are shown in Figure 2.

**ChemRICH analysis.** The 12HA-AID*-TgGS27 and TgTrs85-mAID-3HA strains were cultured in presence or absence of IAA for 24 h. The triplicate samples for each condition were freshly lysed by passing them through 27 G needles and were purified by passing them through a 5-μm-pore filter. The samples were analyzed based on UHPLC-HRMS/MS-based non-targeted lipidomics platform. Briefly, the processes included sample preparation, UHPLC-HRMS/MS analysis, raw mass spectral data preprocessing, univariate and multivariate statistical analysis, and identification of differential lipids. For structural identification of metabolites, the accurate m/z of precursors and product ions were matched against online databases including mzCloud, Metlin, HMDB, MassBank, and local LipidBlast and in-house standard library including retention time, accurate precursors, and product ions. The data was analyzed by ChemRICH software (http://chemrich.fiehnlab.ucdavis.edu).

### **Statistical analysis.** Two-tailed Student’s t-test or one-way analysis of variance was used for statistical analysis. The p values are denoted as follows: *, p ≤ 0.05; **, p ≤ 0.01; ***, p ≤ 0.001; ****, p ≤ 0.0001; and ns, nonsignificant. All data were analysed with GraphPad Prism software (GraphPad Software, San Diego, CA, USA).
