# Supplementary material for: The Dissection of SNAREs Reveals Key Factors for Vesicular Trafficking to the Endosome-like Compartment and Apicoplast via the Secretory System in Toxoplasma gondii
Source: mBio. 2021 Aug 3;12(4):e01380-21. doi: 10.1128/mBio.01380-21 (PMC8406237; doi:10.1128/mBio.01380-21)
Supplement: TABLE S1 [file mbio.01380-21-st001.docx]

| **Name** | ***T. gondii* GT1** | **Type** | **Tag** | **The predicted localization** |
| --- | --- | --- | --- | --- |
| **TgStx4** | **TGGT1_209820** | **Qa** | **TgStx4-ddFKBP-HA** | **Plasma membrane** |
| **TgStx5** | **TGGT1_226600** | **Qa** | **TgStx5-ddFKBP-HA** | **Golgi** |
| **TgStx6** | **TGGT1_300240** | **Qc** | **EGFP-TgStx6** | **Inner membrane complex and trans-Golgi network (TGN)** |
| **TgStx10** | **TGGT1_300290** | **Qc** | **12HA-AID*-TgStx10** | **Endosome-like compartments (ELCs)** |
| **TgStx11** | **TGGT1_220190** | **Qa** | **TgStx11-ddFKBP-HA** | **Lysosome-like vacuolar compartment (VAC)** |
| **TgStx12** | **TGGT1_204060** | **Qa** | **12HA-mAID-TgStx12** | **ELCs** |
| **TgStx16** | **TGGT1_247930** | **Qa** | **6HA-AID*-TgStx16** | **TGN** |
| **TgStx18** | **TGGT1_267530** | **Qa** | **12HA-AID*-TgSTX18** | **ER arrival sites (ERAS)** |
| **TgStx19** | **TGGT1_208010** | **Qc** | **12HA-AID*-TgStx19** | **ERAS** |
| **TgStx20** | **TGGT1_253360** | **Qc** | **12HA-AID*-TgStx20** | **TgCentrin2 annuli** |
| **TgStx21** | **TGGT1_306640** | **Qb** | **12HA-AID*-TgStx21** | **TgCentrin2 annuli** |
| **TgBet1** | **TGGT1_205030** | **Qc** | **3HA-TgBet1** | **Golgi** |
| **TgGS27** | **TGGT1_223620** | **Qb** | **12HA-AID*-TgGS27** | **Golgi** |
| **TgGS28** | **TGGT1_251710** | **Qb** | **12HA-AID-TgGS28** | **Golgi** |
| **TgVtila-1** | **TGGT1_242080** | **Qb** | **TgVtila-1-ddFKBP-HA** | **Plasma membrane** |
| **TgVtilb** | **TGGT1_278160** | **Qb** | **TgVtilb-ddFKBP-HA** | **ELCs** |
| **TgSec20** | **TGGT1_217780** | **Qb** | **3HA-TgSEC20** | **Golgi** |
| **TgSNAP-25** | **TGGT1_319940** | **Qb & Qc** | **TgSNAP25-ddFKBP-HA** | **Cytoplasma** |
| **TgVAMP4-1** | **TGGT1_248100** | **R** | **12HA-AID*-TgVAMP4-1** | **TGN** |
| **TgVAMP4-2** | **TGGT1_246610** | **R** | **12HA-AID*-TgVAMP4-2** | **Apicoplast** |
| **TgVAMP7** | **TGGT1_230430** | **R** | **12HA-mAID-TgVAMP7** | **ELCs** |
| **TgVAMP8** | **TGGT1_257520** | **R** | **TgVAMP8-HA** | **Plasma membrane** |
| **TgYkt6-1** | **TGGT1_215420** | **R** | **TgYKT6-1-ddFKBP-HA** | **Cytoplasma** |
| **TgYkt6-2** | **TGGT1_299180** | **R** | **TgYKT6-2-ddFKBP-HA** | **Cytoplasma** |
| **TgSec22b** | **TGGT1_270070** | **R** | **3HA-TgSEC22b** | **Golgi** |
| **Name** | ***T. gondii* GT1** | **Subunit group** | **Yeast Trs/Bet**  **homologues (Identity** | **Human TrappC/TPC homologues (Identity)** |
| **TgTrs23** | **TGGT1_293050** | **Core TRAPP** | **SGD:S000002654 (26.24%)** | **NP_001305419.1 (26.97%)** |
| **TgTrs31** | **TGGT1_269140** |  | **SGD:S000002880 (22.26%)** | **NP_001035926.1.(39.14%)** |
| **TgBet3** | **TGGT1_299200** |  | **SGD:S000001776 (41.24%)** | **NP_001257823.1 (48.21%)** |
| **TgBet5** | **TGGT1_247648** |  | **SGD:S000004542(14.11%)** | **NP_067033.1 (20.32%)** |
| **TgTrs20** | **TGGT1_222920** | **Adaptor** | **SGD:S000000458 (25.56%)** | **NP_001305419.1 (45.14%)** |
| **TgTca17** | **TGGT1_314720** |  | **SGD:S000000774 (15.38%)** | **NP_001305453.1 (24.10%)** |
| **TgTrs33** | **TGGT1_209080** | **TRAPPI-associated** | **SGD:S000005641 (15.90%)** | **XP_016882784.1 (19.91%)** |
| **TgTrs65** | **TGGT1_312140** | **TRAPPII-specific** | **SGD:S000003398 (8.57%)** | **NP_001087225.1 (6.36%)** |
| **TgTrs120** | **TGGT1_268330** |  | **SGD:S000002815 (5.41%)** | **NP_001361611.1 (5.51%)** |
| **TgTrs130** | **TGGT1_227610** |  | **SGD:S000004831 (3.95%)** | **XP_011528027.1 (4.57%)** |
| **TgTrs85** | **TGGT1_214610** | **TRAPPIII-specific** | **SGD:S000002515 (2.97%)** | **XP_006722483.1 (7.77%)** |

**Supplementary Table 1. List of *T. gondii* SNARE proteins and TRAPP complexes subunits**
