# Supplementary material for: The Dissection of SNAREs Reveals Key Factors for Vesicular Trafficking to the Endosome-like Compartment and Apicoplast via the Secretory System in Toxoplasma gondii
Source: mBio. 2021 Aug 3;12(4):e01380-21. doi: 10.1128/mBio.01380-21 (PMC8406237; doi:10.1128/mBio.01380-21)
Supplement: TABLE S2 [file mbio.01380-21-st002.docx]

**Supplementary Table 2. The antibodies/antisera used throughout this study are indicated.**

| **Laboratory-prepared antibodies** | **Dilutions** | **Commercial antibodies** | **Code No.** | **Dilutions** |
| --- | --- | --- | --- | --- |
| Mouse anti-TgGRA7 | 1:1000 | Mouse anti-Myc | Cell Signalling Technology, 2276S | 1:1000 |
| Mouse anti-TgSAG2 | 1:2000 | Rabbit anti-Myc | Cell Signalling Technology, 2278S | 1:1000 |
| Rabbit anti-TgSAG2 | 1:2000 | Mouse anti-Flag | Sigma, F3165 | 1:1000 |
| Rabbit anti-TgβTubulin | 1:1000 | Rabbit anti-Flag | Cell Signalling Technology, 14793S | 1:1000 |
| Rabbit anti-TgIMC | 1:500 | Mouse anti-HA | Sigma, H9658 | 1:1000 |
| Rabbit anti-TgMIC2 | 1:500 | Rabbit anti-HA | Cell Signalling Technology, 3724S | 1:1000 |
| Rabbit anti-TgM2AP | 1:500 | Mouse anti-V5 | Invitrogen, 377500 | 1:1000 |
| Rabbit anti-TgproM2AP | 1:500 | Mouse anti-Ty | Sigma, SAB4800032 | 1:1000 |
| Rabbit anti-TgAMA1 | 1:500 | Anti-Mouse IgG (H+L) Antibody, DyLight™ 680-Labelled | SeraCare Life Sciences,926-68020 | 1:5000 |
| Rabbit anti-TgRON11 | 1:500 | Anti-Rabbit IgG (H+L) Antibody, DyLight™ 680-Labelled | SeraCare Life Sciences,926-68021 | 1:5000 |
| Rabbit anti-TgRON2 | 1:500 | Anti-Mouse IgG (H+L) Antibody, DyLight™ 800-Labelled | SeraCare Life Sciences, 5230-0415 | 1:5000 |
| Rabbit anti-TgROP4 | 1:500 | Anti-Rabbit IgG (H+L) Antibody, DyLight™ 800-Labelled | SeraCare Life Sciences,926-32211 | 1:5000 |
| Rabbit anti-TgCPL | 1:500 | Alexa Fluor™ 488 anti-mouse IgG antibody | Invitrogen, A11001 | 1:1000 |
|  |  | Alexa Fluor™ 488 anti-rabbit IgG antibody | Invitrogen,A11034 | 1:1000 |
|  |  | Alexa Fluor™ 594 anti-mouse IgG antibody | Invitrogen, A11032 | 1:1000 |
|  |  | Alexa Fluor™ 594 anti-rabbit IgG antibody | Invitrogen,A11037 | 1:1000 |
