# Supplementary material for: The Dissection of SNAREs Reveals Key Factors for Vesicular Trafficking to the Endosome-like Compartment and Apicoplast via the Secretory System in Toxoplasma gondii
Source: mBio. 2021 Aug 3;12(4):e01380-21. doi: 10.1128/mBio.01380-21 (PMC8406237; doi:10.1128/mBio.01380-21)
Supplement: TABLE S3 [file mbio.01380-21-st003.docx]

**Supplementary Table 3. Primer sequences used to amplify and sequencing.**

| **Primer name** | **Sequence** |
| --- | --- |
| gRNA-1S | CCACTAGTTCTAGAGCGGCCGTTTAAACGAGCTCCAAGTAAGCAGAAG |
| TgTrs85-gRNA-1aS | CCAACGAGTTGCGTGTCAAACAACTTGACATCCCCATTTAC |
| TgTrs85-gRNA-1aS-2 | GTTTTCTGGTCAGCGTGCGACAACTTGACATCCCCATTTAC |
| TgTrs85-gRNA-2S | TTTGACACGCAACTCGTTGGGTTTTAGAGCTAGAAATAGC |
| TgTrs85-gRNA-2S-2 | TCGCACGCTGACCAGAAAACGTTTTAGAGCTAGAAATAGC |
| gRNA-2aS | CCGTATTACCGCCTTTGAGTGTTTAAACGAGCTGATACCGCTCGCC |
| TgTrs85-AID-F | CTTCGAGTGTCTCGTCCACATTCAAGAAAGGACTGCAGGGGAGAAGAGCGCGTGTCCT |
| TgTrs85-AID-R | CTCGCACGCTGACCAGAAAACTAGTTTGACACGCAACTCGTTGGTAGCGATGAATTCAGGCATAATCTGGAACATC |
| TgTrs85-SEQ-F | TCGCTTCAGAATCTCCCTGC |
| TgTrs85-SEQ-R | GCGAGTCTAAGCGGTTGAGA |
| TgGS27-gRNA-1aS | GGTCCATGGCGAGAAGACTGCAACTTGACATCCCCATTTAC |
| TgGS27-gRNA-2S | CAGTCTTCTCGCCATGGACCGTTTTAGAGCTAGAAATAGC |
| TgGS27-AID-F | GCCAAAAATCAAGCGGTG |
| TgGS27-AID-R | CCGCTTGATTTTTGGCAG |
| TgGS27-SEQ-F | GCTGTCTGCTCATTCCACCT |
| TgGS27-SEQ-R | GCCTTCTCCCCCTCGAAAAT |
| TgGS27-12HA-F | TCCTCGCTGTCTTGTTCTCCCTTTTTCGCGTGTCTGTCAGTCTTCTCGCCATGTACCCGTACGACGTC |
| TgGS27-12HA-R | ATGCAAAGGGGATACAGCGTCGACAGAGACGCCTCGGAAGGTGCCGGGTCAGAGCCACCTCCTCCACC |
| TgStx12-gRNA-2S | GAAGCGAAGAAATCAGCGACGTTTTAGAGCTAGAAATAGC |
| TgStx12-gRNA-1aS | GTCGCTGATTTCTTCGCTTCCAACTTGACATCCCCATTTAC |
| TgStx12-SEQ-F | CTTGTTCTCCCTCGTTTTCT |
| TgStx12-SEQ -R | CTGAAGGAGAGGTGAGAGAG |
| TgStx12-3HA-F | GGCTACCCATACGATGTTCC |
| TgStx12-3HA-R | GGAACATCGTATGGGTAGCC |
| TgStx12-12HA-F | CACAGAGACGAAAAGGGAGATCGACGAGAAGCGAAGAAATCAGCGACCGAATGTACCCGTACGACGTC |
| TgStx12-12HA-R | TACGGCAATCCCGACGTCGCCAGAAGAGGCGTCGCAGGCTGCTGGAAAGAAGAGCCACCTCCTCCACC |
| TgStx18-gRNA-1aS | TTCTGTCACACATGTTCCAGCAACTTGACATCCCCATTTAC |
| TgStx18-gRNA-2S | CTGGAACATGTGTGACAGAAGTTTTAGAGCTAGAAATAGC |
| TgStx18-SEQ-F | GCTGACACAGTGACGTAAAT |
| TgStx18-SEQ-R | GCTCGCTCAAGGAAAACAAA |
| TgStx18-12HA-F | TTCTGCGGCCTCTTCCGGAAACTCTCGCGAGCAGACTGCGCCTCTGGAACATGTACCCGTACGACGTC |
| TgStx18-12HA-R | ACCTGCTGCAGGGCCCTGCAGCAGGACCGGAAGTCGGGTGTTCTGTCACAAGAGCCACCTCCTCCACC |
| TgStx19-gRNA-1aS | CCTTCGCGTTGTCGAAACATCAACTTGACATCCCCATTTAC |
| TgStx19-gRNA-2S | ATGTTTCGACAACGCGAAGGGTTTTAGAGCTAGAAATAGC |
| TgStx19-SEQ-F | GGTCTCGGTCACCAAAAAGA |
| TgStx19-SEQ-R | CAGCGCTTTCAGTGTCTCAG |
| TgStx19-AID-F | AGGACTTCCTCCAGTTTTCGTGGAGTCTTCCACCTTCGCGTTGTCGAAACATGTACCCGTACGACGTC |
| TgStx19-AID-R | CGCCTCTCCGGCGCGCGAGGGGGAGGAGGCGAGGGAGCCAGAGTATCTGCAGAGCCACCTCCTCCACC |
| TgVAMP4-2-gRNA-1aS | CACGATGCGCCGCCTTTCCTCAACTTGACATCCCCATTTAC |
| TgVAMP4-2-gRNA-2S | AGGAAAGGCGGCGCATCGTGGTTTTAGAGCTAGAAATAGC |
| TgVAMP4-2-AID-F | CGCTTCACCGAATAGAACTTCTCTGTTCGCGTTGAGTTCTCTCCCTCACGATGTACCCGTACGACGTC |
| TgVAMP4-2-AID-R | TCTCGATCGCGCGTCCCCCCTCGCGAGCGAGGAGTGCAGGAAAGGCGGCGAGAGCCACCTCCTCCACC |
| TgVAMP4-2-SEQ-F | TGTGCAGGCGTGGATATATGC |
| TgVAMP4-2-SEQ-R | CCAGGCGACGGCCTTCTTGT |
| TgVAMP4-2-PCR2-AID-F | GCCAAAAATCAAGCGGTG |
| TgVAMP4-2-PCR1-AID-R | CCGCTTGATTTTTGGCAG |
| pBS-GRA-TgACP-F | ATCAAGCAAGAAGCTTGATGGGGCTAGCATGGAGATGCATCCCCGCAA |
| pBS-GRA-TgACP-R | TCAGAGATGAGTTTCTGCTCCGCTGTAGCGCTCTTGGCTTTCTC |
| pBS-GRA-TgACP-3myc-F | TATCGAGAAAGCCAAGAGCGCTACAGCGGAGCAGAAACTCATCT |
| pBS-GRA-TgACP-3myc-R | GTCGTACGGATACATTTAGATATCTTACAGATCTTCCTCAGAGAT |
| pBS-GRA-TgATrx2-F | TCAAGCAAGAAGCTTGATGGGGCTAGCATGATGAAGCAATCCTTTTCC |
| pBS-GRA-TgATrx2-R | CTTCAGAGATGAGTTTCTGCTCGTCCTTTCGTTTCG |
| pBS-GRA-TgATrx2-3myc-F | GCTCGAAACGAAAGGACGAGCAGAAACTCATCT |
| pBS-GRA-TgATrx2-3myc-R | GTCGTACGGATACATTTAGATATCTCACAGATCTTCCTCAGAGAT |
| pBS-GRA-TgCPN60-F | GCAAGAAGCTTGATGGGGCTAGCATGCTTTTAGAACCAGA |
| pBS-GRA-TgCPN60-R | CAGAGATGAGTTTCTGCTCTGCCATTGGCATGTCTGGTAC |
| pBS-GRA-TgCPN60-3myc-F | TGATGTACCAGACATGCCAATGGCAGAGCAGAAACTCATCT |
| pBS-GRA-TgCPN60-3myc-R | GTCGTACGGATACATTTAGATATCTCACAGATCTTCCTCAGAGAT |
| pBS-TUB-TgATrx1-Spe I-F | GAATTCGATGGGGGATCCACTAGTCGACCCATCGAAGACGATCACTGCG |
| pBS-TUB-TgATrx1-Nhe I-R | AAACATGGACCGTGTCTCATGCTAGCTTTAGATCTAAAAGGGAATTCAAG |
| pBS-GRA-TgATrx1-3myc-F | GGAAAGAAATTCTTTGGCGGTCCCCGAAGAGCAGAAACTCATCT |
| pBS-GRA-TgATrx1-3myc-R | GTCGTACGGATACATTTAGATATCTTACAGATCTTCCTCAGAGAT |
| TgAPT1-pro-F | GCGGTGGCGGCCGCTCTAGATTGCCCCCTAACGCAAAA |
| TgAPT1-R | TTCGGTATAGGTTTTCCCATTCTAGATCCGTACTTGGTCT |
| TgFtsH1-pro-F | GCGGTGGCGGCCGCTCTAGATGCTACATCCACAAGAAC |
| TgFtsH1-pro-R | TTCGGTATAGGTTTTCCCATGTGGTCGAAGAGAAAACT |
| TgFtsH1-F | TGGATAGCACACAGCAACAAATGAGTTCTCGTAACGGC |
| TgFtsH1-R | TCGTCGTAGTCctaGATATCTTCGTCTCGCGTAATAGT |
| TgGalNac-F | ATTCCCTTTTAGATCTAAAATGGATGACTCCTCAGAC |
| TgGalNac-R | TGAGTTTCTGCTCGATATCCGGAACAAATACAAAGTGCT |
| TgGalNac-MF | CACTTTGTATTTGTTCCGGATATCGAGCAGAAACTCATCTCT |
| TgGalNac-MR | GCATCACTTTCGTCGTAGTCCTAGATATCCAGATCTTCCTCAGAGAT |
| TgGRASP-F | AGCAAGAAGCTTGATGGGATGGGCTCCTCGC |
| TgGRASP-R | TTCTTCAGAGATGAGTTTCTGCTCGATATCGAGCTCTGGCAAGCCGACCC |
| TgHP03-F | GAAGCTTGATGGGGATATCATGGCGTCCTCGGACTCG |
| TgHP03-R | TCAGAGATGAGTTTCTGCTCAGCGGAGTCTTGCGGTGG |
| pBS-GRA-TgRab5A-F | AAGAAGCTTGATGGGGATATGAGAGGTTTCGAATCT |
| pBS-GRA-TgRab5A-R | AGAGATGAGTTTCTGCTCACTTTTGCCTCCACATGC |
| TgSORTLR-ddGFP-F | ATTCCCTTTTAGATCTAAAATGTACACCTCGCGCACAC |
| TgSORTLR-ddGFP-R | TTTCCACCTGCACTCCCATAAGAAGTTCGACGTTATCCT |
| TgAP1-ddGFP-F | AATTCCCTTTTAGATCTAAAATGGCGGGGGCGTCTGCG |
| TgAP1-ddGFP-R | GTTTCCACCTGCACTCCCATGGAGAGTCTCAGTTGGTA |
| TgRab1B-pro-XbaI-F | CCACCGCGGTGGCGGCCGCTCTAGAGGCATCCCTACCAGAGAG |
| TgRab1B-pro-SpeI-R | GTCGTATTCAGGCTTCATACTAGTGGTTCAAATGCTCGACGA |
| TgRab1B-pro-3myc-XbaI-F | CCACCGCGGTGGCGGCCGCTCTAGAGGCATCCCTACCAGAGAG |
| TgRab1B-pro-3myc-NheI-R | GATCTTCTTCAGAGATGAGTTTCTGCTCGCAACAACCCGAAGAGACGC |
| pBS-GRA-TgStx12-NheI-3myc-F | CAGAAGCTCATCTCTGAGGAAGATCTGATGTCTTTCCAGCAGCCT |
| pBS-GRA-TgStx12-NheI-3myc-R | GGTAACGTCGTACGGATACATGCTAGCCTAGTTCGGACTCTGGGGCAG |
| pBS-GRA-3myc-TgStx10-NheI-F | GAAGCTCATCTCTGAGGAAGATCTGATGGACTTGTGGGGAAGAG |
| pBS-GRA-3myc-TgStx10-NheI-F | ACGTCGTACGGATACATCTAGCTAGCTTACGCGGTCGCCACGACGAG |
| TgStx5-NheI-F | GATCCCCTGGACGCTGCTAGCATGCCGTGCGACAGAACGG |
| TgStx5-EcoRV-R | GCGCATCACTTTCGTCGTAGTCGATATCTCAGCTCAAGAAAAAGACGAAG |
| pBS-TUB-Bet1-3V5-F | TGTTGGGCCTGGACTCCACGATGAGAAAGAAAGAAGGGT |
| pBS-TUB-Bet1-3V5-R | ATCACTTTCGTCGTAGTCGATATCCTAGGCAGACGATTTACCAT |
| pBS-TUB-3V5-TgStx12-F | TCTGTTGGGCCTGGACTCCACGATGTCTTTCCAGCAGCCT |
| pBS-TUB-3V5-TgStx12-R | CGCATCACTTTCGTCGTAGTCGATATCCTAGTTCGGACTCTGGGGCAG |
| TgRab5a-SEQ-F | CAAAGAGCACGATCCGCATG |
| TgRab5a-SEQ-F2 | GGCTGTACGTATGCATGTGC |
| TgRab5a-SEQ-R | ACAGCGAGCAACACAGAGAA |
| TgRab5a-SEQ-R2 | GGGCAGAGAGAGAAAGACGG |
| TgRab5a-3myc-R | CGCAAGAACAGAAGTCTTCTTCGAGCTCGTGGTGTGCATGTGGAGGCAAAAGTGAGCAGAAACTCATCTC |
| TgRab5a-3myc-R2 | AGCCGAGAAACAGTTCTTACAGAAACAGGAGGTTCGAACAAACGAAAGCTTCACAGATCTTCCTCAGA |
| TgSTX10-SEQ-F | CCAATTCTTTTCTCCGCGTCC |
| TgSTX10-SEQ-R | GGGGAAGACAAGGCGATAGG |
| Nhe I-SAG1-HDEL-F | GAGTTTGCCGGGGCTGCAGCTAGCGTGAGCAAGGGCGAGGAG |
| EcoR V-SAG1-HDEL-R | CATCACTTTCGTCGTAGTCGATATCCTACAACTCGTCGTGCTTGTACAGCTCGTCCAT |
| EcoR V-3MYC-HDE2-F | AAGAAATCAAGCAAGAAGCTTGATGGGATGATGGCGATGAATGCCTTTCGT |
| EcoR V-3MYC-HDE2-R | ATCTTCTTCAGAGATGAGTTTCTGCTCCACTTCTGCGTGGACGGGCAACA |
| SEC13-3V5-F | CAAGCAAGAAGCTTGATGGGCTAGCATGGCGGCTCCAGCGACGCT |
| SEC13-3V5-R | GAGGGGGTTCGGTATAGGTTTTCCGTTTCCTTTGTAAGGCGCATAC |
